# Nitric oxide signaling in ctenophores

**DOI:** 10.3389/fnins.2023.1125433

**Published:** 2023-03-22

**Authors:** Leonid L. Moroz, Krishanu Mukherjee, Daria Y. Romanova

**Affiliations:** ^1^Department of Neuroscience, McKnight Brain Institute, University of Florida, Gainesville, FL, United States; ^2^The Whitney Laboratory for Marine Bioscience, University of Florida, St. Augustine, FL, United States; ^3^Institute of Higher Nervous Activity and Neurophysiology of RAS, Moscow, Russia

**Keywords:** *Mnemiopsis*, Ctenophora, nitric oxide synthase, guanylate cyclase, *Pleurobrachia*, nervous system evolution, Porifera, Placozoa

## Abstract

Nitric oxide (NO) is one of the most ancient and versatile signal molecules across all domains of life. NO signaling might also play an essential role in the origin of animal organization. Yet, practically nothing is known about the distribution and functions of NO-dependent signaling pathways in representatives of early branching metazoans such as Ctenophora. Here, we explore the presence and organization of NO signaling components using *Mnemiopsis* and kin as essential reference species. We show that NO synthase (NOS) is present in at least eight ctenophore species, including *Euplokamis* and *Coeloplana*, representing the most basal ctenophore lineages. However, NOS could be secondarily lost in many other ctenophores, including *Pleurobrachia* and *Beroe*. In *Mnemiopsis leidyi*, NOS is present both in adult tissues and differentially expressed in later embryonic stages suggesting the involvement of NO in developmental mechanisms. Ctenophores also possess soluble guanylyl cyclases as potential NO receptors with weak but differential expression across tissues. Combined, these data indicate that the canonical NO-cGMP signaling pathways existed in the common ancestor of animals and could be involved in the control of morphogenesis, cilia activities, feeding and different behaviors.

## Introduction

A free radical gas, nitric oxide (NO), is an evolutionary old and versatile signal molecule with a widespread distribution across all domains of life (Feelisch and Martin, 1995; Moroz and Kohn, 2011; Shepherd et al., 2022). NO can be synthesized by numerous non-enzymatic pathways (Moroz and Kohn, 2011) and enzymatically as parts of the nitrogen cycle and by NO synthases (NOS). NOSs were identified both in prokaryotes and eukaryotes. All NOSs catalyze the oxidation of L-arginine by molecular oxygen with several co-factors required for electron transfer from NADPH *via* FMN in the reductase domain to heme-/biopterin binding sites of the N-terminal oxygenase domain (Stuehr and Haque, 2019). Prokaryotic NOSs are usually truncated, often without the reductase part, and might contain additional subdomains (e.g., globin-like). Animal NOSs always have Ca^2+^/calmodulin-binding domains that tightly control the electron transport and NO yield in response to environmental stimuli (Stuehr and Haque, 2019).

Eukaryotic type NOS was recently discovered in *Salpingoeca infusionum*, the representative of Choanoflagellata (Reyes-Rivera et al., 2022) – the sister group to Metazoa. In another choanoflagellate *Choanoeca flexa*, with prokaryotic-type NOS, NO application induced contractions of colonies, providing a shift from feeding to swimming behaviors. Such an effect can be mediated by soluble guanylate cyclase (sGC), as in animals (Reyes-Rivera et al., 2022). These data and the presence of NOS in other eukaryotes suggest that the common ancestor of all animals might possess NOS-sGC signaling pathways. However, little is known about the distribution and functions of NO-mediated (nitrergic) signaling in early branching metazoans (Colasanti et al., 2010).

There are five major animal clades: Bilateria, Cnidaria, Placozoa, Porifera (sponges), and Ctenophora (comb jellies). The last four groups represent the earliest branching animal lineages, separated from Bilaterians more than 550 million years ago. These non-bilaterians are remarkably different in their bodyplans and tissue organizations. As a result, they are crucial to reconstruct the evolution and overall architecture of animal signaling pathways, including NO-dependent transmission. Yet only several papers deal with NO biology in Cnidaria and Porifera; and one publication discusses NO synthesis in placozoans (Moroz et al., 2020).

Ca-dependent and heat-stress-induced NO synthesis was reported in two species of desmosponges, *Axinella polypoides* and *Petrosia ficiformis* (Giovine et al., 2001). NADPH diaphorase histochemistry [a marker for NOS (Bredt et al., 1991; Moroz, 2000a)] showed specific localization of NOS activity in a particular network of dendritic cells in the sponge parenchyma (Giovine et al., 2001), but with unknown functions.

NO, and NOS control metamorphosis and symbiotic relationships in the desmosponge *Amphimedon* (Ueda et al., 2016; Hewitt and Degnan, 2022). There is growing evidence that NO can modulate body contractions and coordinated behaviors in freshwater sponges *Ephydatia muelleri* (Elliott and Leys, 2010), *Spongilla lacustris* (Musser et al., 2021), and the marine sponge *Tethya wilhelma* (Ellwanger and Nickel, 2006).

Similarly to sponges, NO signaling was implemented in cnidarian-algal symbioses (Perez and Weis, 2006; Safavi-Hemami et al., 2010; Hawkins and Davy, 2013), coral bleaching (Bouchard and Yamasaki, 2008), and apoptosis (Hawkins et al., 2013). In the hydroid polyp *Hydra*, non-neuronal NO/NOS is associated with regeneration (Colasanti et al., 2009) and feeding (Colasanti et al., 1995). Specific nitrergic neurons have been identified in tentacles of the hydromedusa *Aglantha digitale*, where the NO/cGMP pathway modulates the rhythmic swimming associated with feeding (Moroz et al., 2004). Multiples isoforms of NOSs present in the majority of sequenced cnidarian genomes, with evidence of endogenous enzymatic NO synthesis in this lineage (Morrall et al., 2000; Moroz et al., 2004; Kass-Simon and Pierobon, 2007; Cristino et al., 2008; Anctil, 2009; Colasanti et al., 2010).

The overall logic of NO signaling in early animals is still elusive. Nevertheless, both in sponges and cnidarians, we might expect tightly coupled interplays of morphogenic and behavioral functions mediated by NO, inherently linked to the ancestral feeding modes and innate immunity (Moroz, 2000b,^2001^). Scattered comparative data point out that the volume transmission mediated by NO in nervous systems was a relatively later innovation in evolution, (Moroz et al., 2021). Unfortunately, no data are available about NOSs and NO signaling in ctenophores or comb-jellies with well-developed neuro-muscular organization across species (Norekian and Moroz, 2016, 2019a,b, 2020, 2021).

Ctenophora is one of the earliest branching lineages of metazoans with complex tissue and organ differentiations. All extant ctenophore species have well-developed neuronal and muscular systems, which might co-evolve independently (Moroz, 2015). The recently proposed most basal position of ctenophores (as the sister group to all other animals) is still highly debated (Whelan et al., 2015, 2017; Halanych et al., 2016; Telford et al., 2016; Laumer et al., 2019; Fernandez and Gabaldon, 2020; Kapli and Telford, 2020; Li et al., 2021; Redmond and McLysaght, 2021; Giacomelli et al., 2022). Nevertheless, the presence or absence of specific signaling pathways in the ctenophore lineage reshapes our general understanding of neuronal and animal evolution. NOS was not detected in the sequenced genome of *Pleurobrachia bachei* (Moroz et al., 2014), but we found a putative NOS in *Mnemiopsis leidyi* (Moroz and Kohn, 2016) by screening its sequenced genome (Moreland et al., 2020). Here, we further explore the presence and organization of NO signaling components using *Mnemiopsis* as essential reference species (Moreland et al., 2020).

## Results and discussion

### NOS phylogeny and derived NOS in ctenophores

**FIGURE 1:**
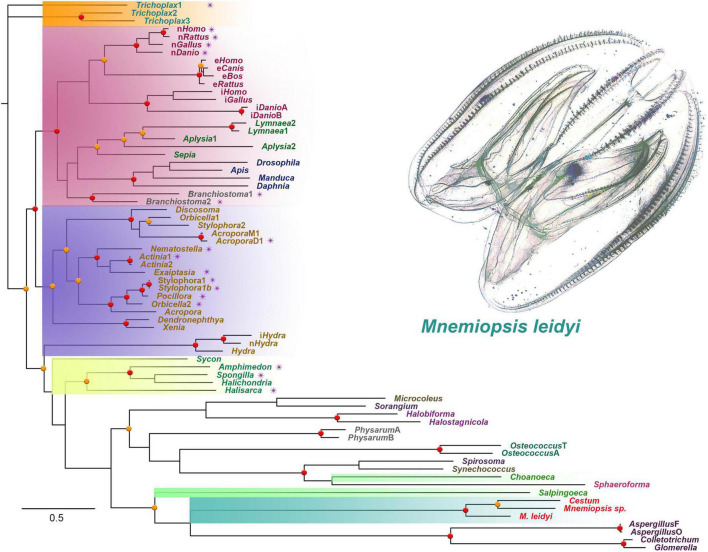
The phylogenetic tree of nitric oxide synthases (NOS) across different taxa, focusing on representative metazoan clades (ML analysis). Major taxons are highlighted: Ctenophora – aquamarine; Porifera – yellow; Placozoa – orange; Cnidaria – blue; Vertebrata/Bilateria – red; Choanoflagellata – bright green. The presence of PDZ domains is marked as purple stars near the species names. Bootstraps: red (90–100%) and orange (80–89%) dots.

Figure 1 shows the phyletic relationships among basal metazoan NOSs and several eukaryotic (e.g., Amoebozoa [*Physarum*], Fungi [*Aspergillus* and kin], and Ichthyosporea [*Sphaeroforma arctica*]) and prokaryotic outgroups. This tree confirms a highly derived nature of NOS in ctenophores, probably reflecting their accelerated evolution and a possible bottleneck in their paleontological history around the Permian extinction (Whelan et al., 2017). The tree also highlights several independent radiation events with duplication and triplication of NOSs in lineages leading to placozoans, cnidarians, and vertebrates [see also (Moroz et al., 2020)]. However, representative species from both Porifera and Ctenophora have only one NOS gene.

In addition to the sequenced ctenophore genomes (*Pleurobrachia bachei* and *Mnemiopsis leidyi*), we analyzed transcriptomes from 37 ctenophore species (Whelan et al., 2017). NOSs were found only in eight ctenophores, including six representatives of Lobata: *Mnemiopsis leidyi*, undescribed species of *Mnemiopsis* sp., and their sister species *Bolinopsis infundibulum* [the family Bolinopsidae], the venus girdle, *Cestum veneris* [Cestidae], *Ocyropsis crystallina* [Ocyropsidae], *Lobatolampea tetragona* [Lobatolampeidae]. Most interesting, NOS was found in *Euplokamis dunlapae* [Euplokamididae] and the benthic ctenophore *Coeloplana astericola* [Platyctenida, Coeloplanidae] – representatives of the first and second earliest branching lineages within the phylum Ctenophora (Moroz et al., 2014). In contrast, *Bolinopsis*, *Mnemiopsis, Cestum, Ocyropsis, Lobatolampea* belong to separate branches within a highly derived (and possibly polyphyletic) clade of Lobata (Whelan et al., 2017).

NOS-type sequences were not identified in more than two dozen transcriptomes from adult tissues and different developmental stages from *Pleurobrachia*. Moreover, NOS was not found in the recently sequenced genome of a closely related species *Hormiphora californica* [Pleurobrachiidae] (Schultz et al., 2021). These data suggest that the majority of sequenced so far ctenophore lineages (including Cydippida and Beroida) lost NOS from their common ancestors. This apparently massive loss of NOS in many representatives of Ctenophora is quite unusual compared to other phyla. For example, NOS was lost in some nematodes such as *C. elegans*, but for most animal lineages, NOS genes are well-preserved, despite enormous ecological and morphological diversifications.

### Domain organization of NOS in ctenophores

**FIGURE 2:**
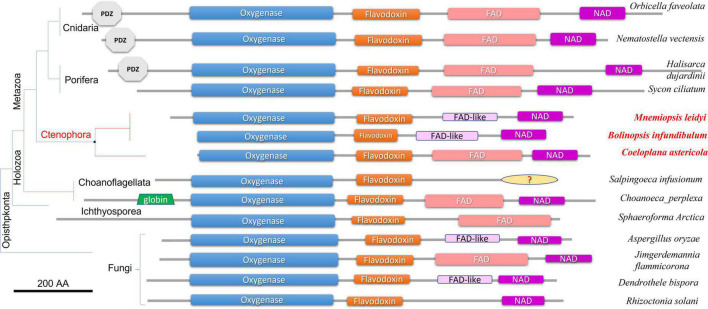
Domain organization of NOSs across eukaryotic phyla. All NOS are presented on the same scale, including the sizes of all domains and proteins. NOS oxygenase domain (Pfam NO_Synthase PF02898); Flavodoxin_1 (Pfam PF00258); FAD_binding_1 domain (Pfam PF00667); NAD_binding_1 (Pfam PF00175). NOSs include representatives of 3 metazoan phyla and 3 non-metazoan lineages with their respective phylogenetic relationships and species names. The references for each particular gene and/or their sequences with relevant GeneBank accession numbers are summarized in the Supplementary Table 1.

Figure 2 illustrates the domain organization of NOS across species. The *Coeloplana*, *Bolinopsis*, and *Mnemiopsis* NOSs revealed the canonical basal domain architecture of this class of enzymes (Figure 2). At the same time, sequences from other ctenophore species were represented mainly by the oxygenase domain and were incomplete genes, which the nature of transcriptome datasets can explain. Thus, we used *M. leidyi* NOS (ML074215a) as the reference sequence for these types of enzymes in ctenophores.

At least three features distinguish ctenophore NOSs from many other species. First, both in *Bolinopsis* and its sister species *Mnemiopsis*, a critical for NOS function FAD domain is either highly derived or might need to be better recognized. Still, their NOSs perfectly preserved NAD-binding sites (Figure 2). With this architecture NOSs from *Bolinopsis* and *Mnemiopsis* are similar to eukaryotic NOSs from the choanoflagellate *Salpingoeca infusionum*, and some fungi such as *Aspergillus* (Ascomycota) and *Dendrothele* (Basidiomycota). Highly modified or derived FAD-binding domains in these species might affect complex or unusual electron transport mechanisms from NADPH to heme in NOS (Stuehr and Haque, 2019). Yet, such FAD-related ctenophore modifications are likely secondarily derived. The canonical FAD-binding region is well-conserved in the benthic ctenophore *Coeloplana* (Figure 2), which probably represents the ancestral condition for ctenophore NOSs.

Second, *Mnemiopsis leidyi* NOS lacks the PDZ domain, found in many NOSs from poriferans (desmosponges *Halisarca*, *Spongilla*, and *Amphimedon*, but not from the calcareous sponge *Sycon ciliatum*), Placozoa (*Trichoplax*), cnidarians (corals, sea anemones), and bilaterians/chordates (Figures 1, 2). PDZ domain is best studied in mammals and is responsible for anchoring neuronal NOSs in specific membrane compartments and protein complexes, facilitating more localized and spatial control of NO release and signaling in synapses (Brenman et al., 1996; Stricker et al., 1997; Jaffrey et al., 1998; Kim and Sheng, 2004; Feng and Zhang, 2009; Sheng et al., 2018; Murciano-Calles et al., 2020). Of note, PDZ NOSs were not found in non-metazoan eukaryotes and prokaryotes and can be viewed as animal innovation coupled with their morphological and signaling complexities.

**FIGURE 3:**
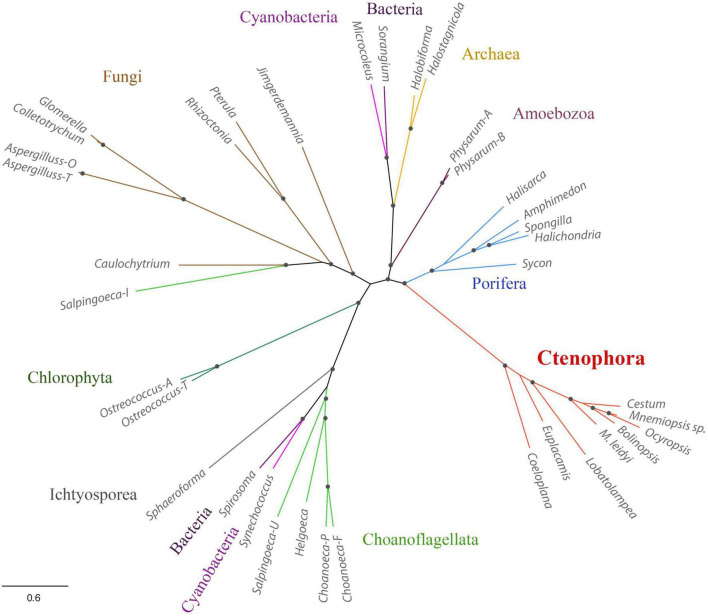
Phylogenetic relationships of NOSs in ctenophores, sponges, non-metazoan eukaryotes, and prokaryotes. The references for each particular gene and/or their sequences with relevant GeneBank accession numbers are summarized in the Supplementary Table 1.

Third, *Mnemiopsis leidyi* NOS is shorter or truncated than known cnidarian, poriferan, and placozoans NOSs. In this case, it is also superficially similar to non-metazoan NOSs. Yet ctenophore NOSs make a highly derived branch on the phylogenetic trees with different representations of species (Figures 1, 3), suggesting an accelerated evolution within this group. A similar situation is also observed for choanoflagellates. Most choanoflagellate species apparently lose their NOSs from their common ancestor. The eukaryotic type of NOS is only preserved in one species, *Salpingoeca infusionum*, which is clustered with fungal NOSs (Figure 3) and lack some evolutionarily conserved regions, such as FAD- and NAD-binding (Figure 2). Other four choanoflagellate species had prokaryotic-type NOS [see (Reyes-Rivera et al., 2022) and Figures 2, 3]; these NOS might be results of a horizontal gene transfer from cyanobacteria.

**FIGURE 4:**
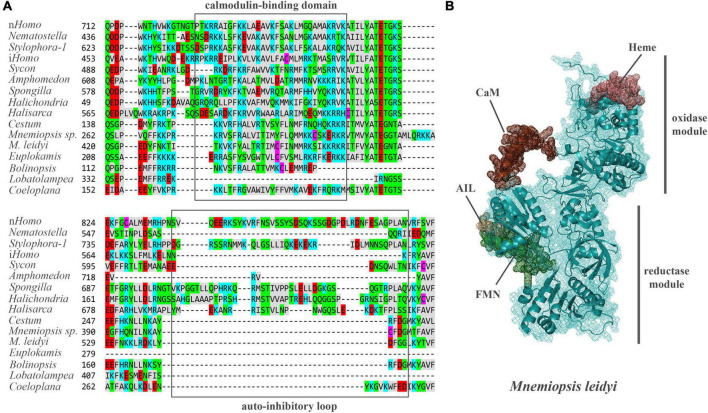
**(A)** The structure of auto-inhibitory loop and calmodulin-binding domains in NOSs. Amino acids (aa) are highlighted with different colors: red – polar negative charged aa; blue – polar positive charged aa; purple – cysteines; gray – hydrophobic aa; green – uncharged polar aa. **(B)** The predicted 3D structure of the NOS from *Mnemiopsis leidyi* with critical functional domains. Calmodulin (CaM) binding – brown, FMN – green, AIL – beige. The Supplementary Table 1 summarizes the references for each particular gene and/or their sequences with relevant GeneBank accession numbers.

*Mnemiopsis leidyi* NOS (ML074215a) has a recognized Ca/Calmodulin-binding region coupling oxygenase and reductase domains as in all animal NOSs (Figure 4, see Supplementary material for alignment), suggesting calcium-calmodulin dependence. In mammals, transient activation of NOS by intracellular Ca^2+^ is controlled by the auto-inhibitory loop/inserts (Roman and Masters, 2006; Stuehr and Haque, 2019), without sequence similarities in neuronal and endothelial isoforms. In contrast, inducible Ca-independent NOS lacks this sequence motif (Figure 4A). Thus, activation of its expression is induced by bacterial liposaccharides as a component of the innate immune defense mechanisms. Interestingly, ctenophore NOSs also lack the autoinhibitory loops (Figure 4A), and their Ca-dependence, enzymology, and expression control must be experimentally characterized. Ca-dependences of NOS in sponges and cnidarians are also unknown. However, both poriferan and cnidarian NOSs show more remarkable overall sequence similarities with their bilaterian homologs.

Of note, we also found bacterial types of NOS in the *Mnemiopsis* microbiome (Mariita et al., 2021), suggesting both endogenous and exogenous sources of NO in ctenophores (e.g., from food or symbionts).

### Soluble guanylyl cyclases as putative receptors of NO in ctenophores

As a free radical gas, NO can interact with most biological molecules, primarily targeting SH and tyrosine groups (Holguín-Peña et al., 2007). However, in animals including cnidarians, placozoans, and sponges, NO can specifically activate soluble guanylyl cyclases [sGC, members of the adenylate cyclase superfamily (Gancedo, 2013; Bassler et al., 2018)] by binding to its heme group, which leads to conformational changes and increase of cGMP synthesis (Krumenacker et al., 2004; Martin et al., 2005; Windsor and Thompson, 2008; Gunn et al., 2012; Montfort et al., 2017; Horst and Marletta, 2018; Horst et al., 2019; Kang et al., 2019). Three orthogroups of sGCs are present in humans.

**FIGURE 5:**
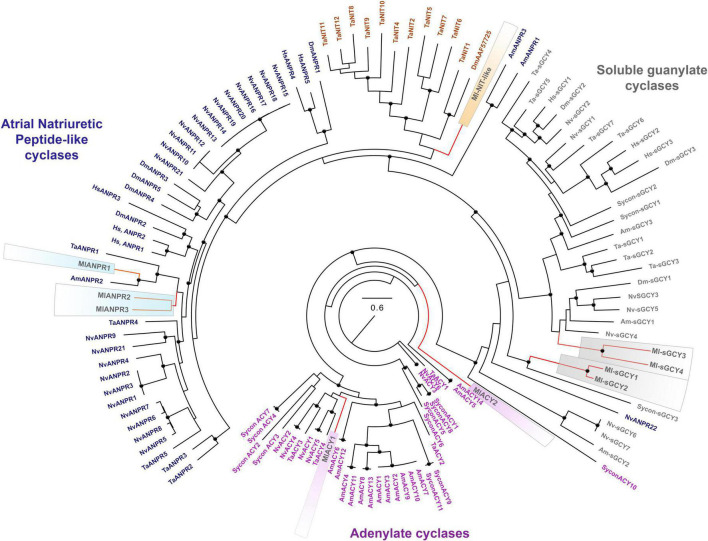
Phylogenomic ML analyses of cyclases across representative metazoans with the focus on *Mnemiopsis leidyi* and kin. Major classes of cyclases are highlighted by purple (adenylate cyclases), blue (ANPRs), brown (containing NIT-like domain cyclases), and gray (soluble guanylate cyclases). Bootstrap values are shown as black dots (90–100%). Genes of *M. leidyi* placed in color frames: purple (for ACYs), blue (for ANPRs), orange (for one putative NIT-like containing domain gene), gray (for sGCYs). The references for each particular gene and/or their sequences with relevant GeneBank accession numbers are summarized in the Supplementary Table 1.

The *Mnemiopsis* and *Pleurobrachia* genomes encode four and two sGCs, respectively (Figure 5 and Supplementary Figure 1). These enzymes have the canonical heme NO binding domain and associated cyclase domain. Moreover, we also identified sGCs in several other ctenophore species with and without detectable NOS (Supplementary Figure 1). For example, there are four sGCs in the lobate *Ocyropsis* with NOS as in *Mnemiopsis*. In contrast, we have a reduced representation of sGCs in *Pleurobrachia* and other Cydippida and Beroida. Thus, the absence of NOS from the genomes does not exclude a possibility of NO signaling due to its non-enzymatic productions by different mechanisms (Moroz and Kohn, 2011) or from exogenous sources (environment, food, microbiomes, etc.).

Recent research also indicates that sGCs can be co-present with NOS in choanoflagellates, controlling swimming behaviors of colonies (Reyes-Rivera et al., 2022) and implying pre-metazoan origins of these signaling pathways. From this apparently simple ancestral condition, sGCs show lineage-specific radiations for many phyla, including 3 sGCs in sponges and humans, seven sGCs in placozoans and cnidarians (Figure 5).

**FIGURE 6:**
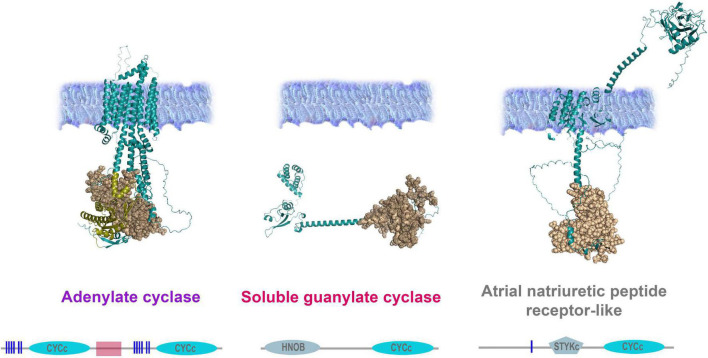
Organization of adenylate and guanylate cyclases in *Mnemiopsis leidyi*. Representative cyclases show notable structural differences: Adenylate cyclase anchor in the membrane with ten transmembrane regions and contain two intracellular catalytic CYCc domains; ANPR-like (atrial natriuretic peptide receptor-like) has the extracellular part (ANF-receptor) and two transmembrane domains, STYKc and CYCc domains are located in the intracellular space; soluble guanylate cyclase is located in the cytoplasm. All sequences were analyzed using SMART (Letunic and Bork, 2018; Letunic et al., 2021), NCBI Conserved Domain Search (Lu et al., 2020), and were built using AlphaFold2 (Jumper et al., 2021). The references for each particular gene and/or their sequences with relevant GeneBank accession numbers are summarized in the Supplementary Table 1.

In addition, ctenophores encode two other classes of membrane-bound cyclase candidates, such as a derived atrial natriuretic peptide receptor [ANPR] type group, probably involved in peptide sensing, and an enigmatic group of cyclases with NIT domains (Figures 5, 6). *Mnemiopsis* has three orthologs of ANPR-like receptors with CYC/cGMP coupling.

Unique NIT (PF08376) domains (Oulavallickal et al., 2017), containing cyclases, were previously identified in *Trichoplax* (Moroz et al., 2020) as candidates for nitrite/nitrate sensors, similar to bacteria (Shu et al., 2003; Camargo et al., 2007). *Mnemiopsis* has one sequence (ML02033a) associated with the same cluster (Figure 5) but with a highly derived NIT-like region and unknown function.

Finally, *Mnemiopsis* has two adenylate cyclases (ML04963a, ML20918a) involved in the evolutionarily conserved cAMP signaling (Figures 5, 6). Surprisingly, the genome screening indicates that cnidarians and sponges possess more adenylate cyclases (5-14) than ctenophores (Figure 5).

### Expression NOS and putative functions of NO in ctenophores

**FIGURE 7:**
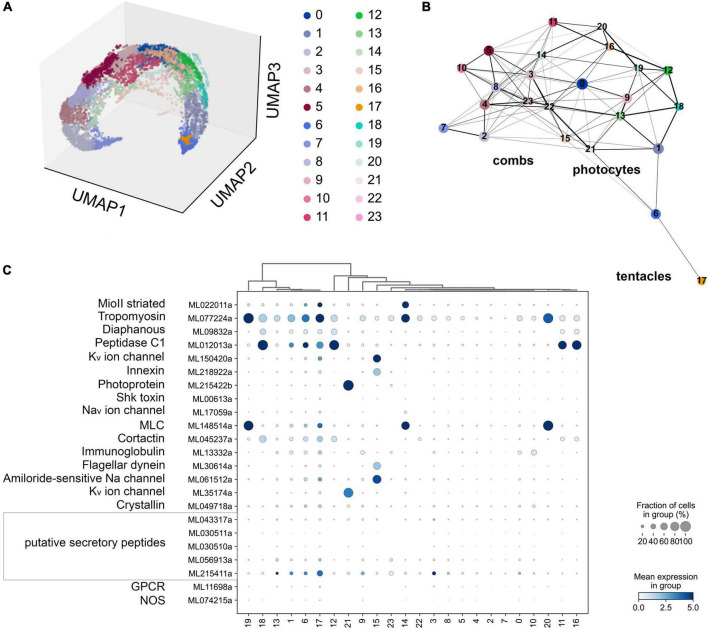
**(A)** The UMAP clustering for 6,144 cells of *Mnemiopsis leidyi* (Sebé-Pedrós et al., 2018) with 24 clusters. **(B)** The PAGA analysis placed clusters with connections based on their differential expressed genes. We found comb associated cluster (#15) using innexin, and flagellar dynein marker accordingly (Sebé-Pedrós et al., 2018). Photocyte’s cluster (#21) can be recognized using such markers as photoprotein and potassium channel. The tentacle cluster #17 is placed separately; it contains both marker genes (secretory peptides, tropomyosin, potassium and sodium channels (Sebé-Pedrós et al., 2018)) and putative NOS expressing cells. **(C)** Dotplots highlights cluster-specific genes (Sebé-Pedrós et al., 2018; Burkhardt and Jékely, 2021).

**FIGURE 8:**
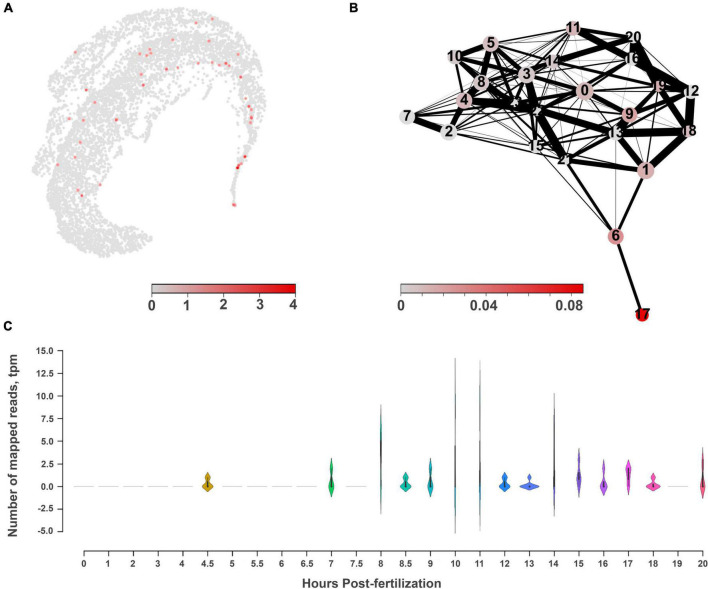
The molecular architecture and distribution of nitric oxide synthase (NOS) in *Mnemiopsis leidyi*. **(A)** The UMAP clustering for NOS shows a relatively diffuse distribution across several cell types with low expression levels. **(B)** PAGA analysis for NOS highlights its expression in cluster #17, which might belong to tentacles, according to the marker genes (Sebé-Pedrós et al., 2018). **(C)** RNA-seq profiling of NOS expression in development based on Levin et al. (2016), Moreland et al. (2020). NOS expression is detected after 7th hour of post-fertilization and is associated with the morphogenesis of major ctenophore organs.

**FIGURE 9:**
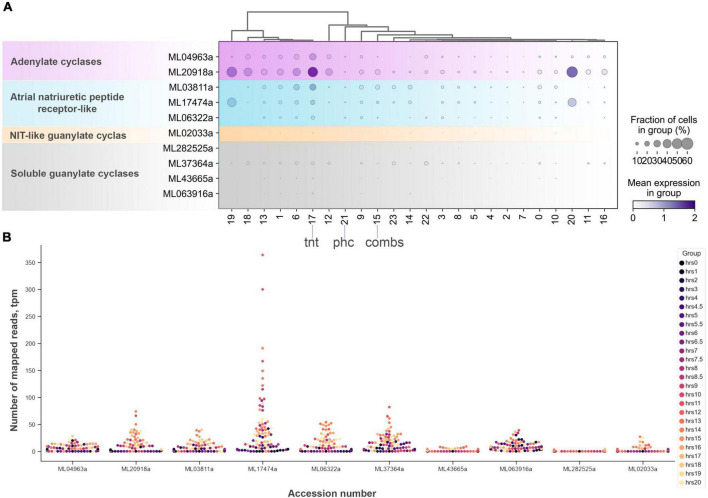
The diversity and expression of cyclase genes in *Mnemiopsis leidyi* based on scRNA-seq profiling (Sebé-Pedrós et al., 2018) (see text for details). **(A)** Dotplot analysis indicates that all cyclases are expressed in cluster #17 (tentacles) except one soluble guanylate cyclase (ML282525a). sGCY (ML37364a) has low expression levels in all cell clusters from an adult animal. However, the majority of sGCYs are expressed in developmental stages **(B)**. RNA-seq profiling of expression in development is based on Levin et al. (2016), Moreland et al. (2020). Post-fertilization hours are indicated as color dots on the right.

Two groups of datasets provide initial estimates of the distribution of NOS and sGC expressions in *Mnemiopsis leidyi* (Figures 7–9): single cells data (scRNA-seq) from adult animals and transcriptomes (RNA-seq) of different embryonic and developmental stages (Levin et al., 2016).

According to the scRNA-seq datasets from 5,461 cells filtered as in Sebé-Pedrós et al. (2018), NOS was a relatively low-expressed gene, present in individual cells more or less randomly distributed in 15 clusters (out 41), including candidate clusters for some secretory cells (recognized with neuropeptide markers identified for this species). We did not detect NOS expression in combs and photocytes.

Next, considering the low abundance of NOS-containing cells and low level of expression, we performed a similar analysis without any filtration with attempts to capture rare cell types. This analysis with 6,144 cells detects a very low level of NOS expression in 15 out of 24 clusters (Figure 7), including a few cells with NOS in tentacle-associated cells/tissues, as also shown with partition-based graph abstraction (PAGA) analysis (Wolf et al., 2019).

Among all identified cyclases, adenylate cyclases and ANP-like receptors with guanylyl cyclases are the most abundantly expressed (Figure 9). Their expression is detected in most cell types, including tentacles and putative neurons. Nevertheless, sGCs are also very weakly expressed, similar to NOS. In summary, a small number of sequenced cells and an extremely low level of detected NOS/sGC expression prevent making definite conclusions about cell type specificity that might employ this signaling pathway. In future studies, this ambitious task would require multi-color *in situ* hybridization and immunohistochemistry experiments. A limited number of ctenophore cell-specific molecular markers also prevent more detailed annotation of these scRNA-seq data.

RNA-seq data from development contain significantly deeper sequencing than single-cell datasets. During development, NOS (ML074215a) expression became noticeable starting with the 7th hour of post-fertilization (pft), and further increased at the 10th and 11th hours, and later decreased. Here, NOS expression was associated with the specification of tissues and formation of ctenophore-specific organs such as combs, the aboral organ, and components of the digestive systems [e.g., (Simmons et al., 2012; Fischer et al., 2014; Reitzel et al., 2016)]. Expression of sGC was also weak in development and partially corresponded to the same or later stages of development compared to NOS expression (Supplementary Figure 2).

**FIGURE 10:**
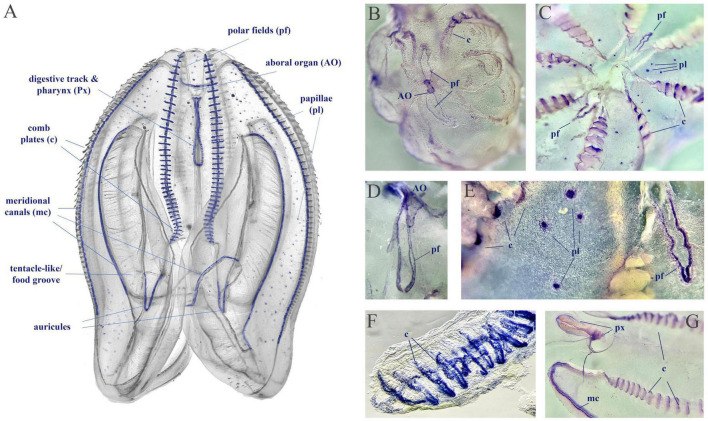
NADPH-diaphorase histochemistry in *Mnemiopsis leidyi*. **(A)** The distribution of putative NOS activity in *Mnemiopsis*. This schematics illustrate the regions with putative NOS, but not reflect quantitative estimates of tentative enzymatic activities. **(B,C)** Whole mount preparation with NADPH-d reactivity in the aboral organ (AO), the polar fields (pf), in comb plate areas (c), and papillae (pl). **(D–G)** Illustrated examples of NADPH-d reactivity across different structures, including the pharynx (px) and the meridional canal (mc) near the auricules (a feeding structure).

We used fixative-resistance NADPH-diaphorase (NADPH-d) histochemistry as a reporter of NOS activity (Bredt et al., 1991; Moroz, 2000a) across phyla, from cnidarians to vertebrates (Bredt et al., 1991; Kurenni et al., 1995; Moroz et al., 2000, 2004; Giovine et al., 2001; Bishop et al., 2008; Moroz and Kohn, 2011). *Mnemiopsis* has extremely fragile tissues, often disintegrated during conventional fixation, highly limiting *in situ* hybridization experiments, requiring multiple steps. However, we managed to perform simpler NADPH-d protocols using larger (2–5 cm) adult animals (*n* = 6). The NADPH-d reactivity (putative NOS activity) is broadly distributed across tissues in *Mnemiopsis* (Figure 10), including cells in the aboral organ, polar fields, base of combs, auricles, and meridional canals regions, as well as putative sensory papillae around the body surface, and in components of the digestive system.

**FIGURE 11:**
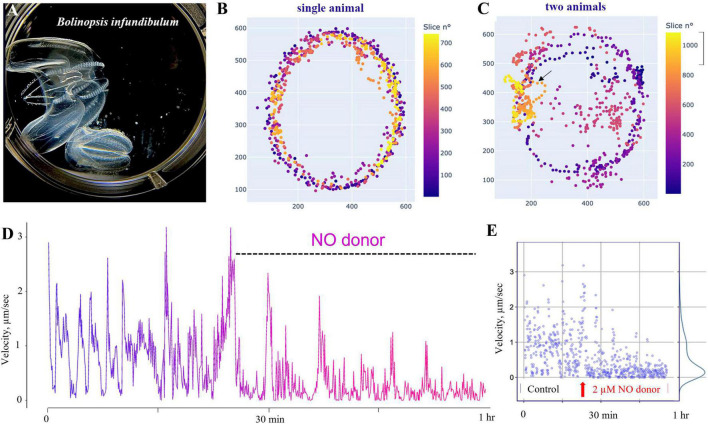
Nitric oxide suppresses swimming in *Bolinopsis.* **(A)** The photo of two *B. influndibulum* in an experimental Petri dish. **(B,C)** Velocity plots (color gradient at right) following the application of 2 μM of DEANO (NO donor). Illustrative tests on a single and two animals in the dish show a decrease of the speed of locomotion and travel distance following NO application. **(D)** Dynamic swimming shows oscillations in the velocity and gradual decay of locomotory speed in the presence of DEANO. **(E)** Velocity ranged from 0 to 3 in the control group, and these values gradually reduced to a nearly complete arrest of swimming in the experimental group of eight animals.

Furthermore, low expression levels do not imply the lack of functional role of NO-cGMP signaling because this pathway includes significant amplification cascades. Thus, we performed pilot pharmacological experiments to test the effects of NO on free-behaving animals (Figure 11).

Specifically, we used *Bolinopsis* as a model for these initial tests. Applications of the NO donor DEA/NO 2–20 μM (*n* = 8 for each concentration) resulted in the suppression of locomotion within the first 10–20 min, including reduction of long-term oscillations of swimming patterns, and eventually the suppression of swimming usually within 60–90 min (*n* = 8). Higher concentrations of the NO donor (20–200 μM) were apparently toxic, leading to a rapid arrest of locomotion and dissociation of animals (*n* = 8). Of note, applying the same concentrations to *Pleurobrachia* and *Beroe* did not result in such immediate toxic effects.

## Discussion: Conclusions and future directions

This manuscript outlines the first survey of NOS distribution and functions in ctenophores. The presence of NOS and intracellular NO receptors such as sGC in ctenophores are supported by molecular evidence. However, both comparative and, especially, functional aspects of NO-mediated signaling remain to be determined, and numerous components of NO/cGMP-mediated signaling can be a particular ctenophore lineage-specific. Conclusions are summarized below.

(1) NOS is present in ctenophores with the events of secondary gene loss across several species, primarily in cydippids, for yet unknown reasons.

(2) 2–4 sGCs are also present in ctenophores, with taxonomically broader distribution than NOSs, including their presence in species without NOS. These observations suggest the occurrence of other NO synthetic pathways [enzymatic and non-enzymatic (Moroz et al., 1998; Moroz and Kohn, 2011) and environmental sources of NO (e.g., from microbiota and symbiotic organisms)]. Of note, sGC can also sense CO and potentially other molecules (Feelisch and Martin, 1995; Moroz and Kohn, 2011; Stuehr and Haque, 2019; Shepherd et al., 2022).

(3) In *Mnemiopsis*, NOS and sGCs are expressed at later stages during embryogenesis, and this pathway might be associated with tissue and organ specification in ctenophore development.

(4) Although NO/sGC showed a relatively low expression level in adults, NO might be involved in both localized and systemic control of locomotion with cilia as one of the potential targets of this signaling (to be experimentally validated).

We would like to stress the importance of studying the non-neuronal and systemic functions of NO in ctenophores. NADPH-d did not reveal labeling of defined neuronal populations in mesoglea or subepithelial neural nets or particular comb areas where neurons were identified in previous studies (Moroz, 2015; Norekian and Moroz, 2016, 2019a,b, 2020, 2021). Although cells at the base of combs or ciliated plates in the auricles were NADPH-d positive, they are unlikely neurons. Similarly, the labelings in the aboral organ (with the gravity sensor) and two other putative sensory structures (polar fields as well as papillae) do not convincingly occur in neurons.

These observations are consistent with our pilot findings about relatively small instant effects of NO on behaviors in two lobate ctenophores. In addition to *Bolinopsis*, we performed similar pilot pharmacological tests on *Mnemiopsis* using the same NO donor (DEA NO, 21 animals). Lower concentrations of NO donors (<1 μM, *n* = 7) had no noticeable effects; higher concentrations (>10–70 μM) moderately suppressed the ciliated locomotion and modulate muscle contractions.

Clearly, more systematic studies have to be performed using different NOS donors, inhibitors, and drugs affecting cGMP signaling using cilia, muscles, and secretory cells as effectors, to name a few. However, those observed and relatively “weak” effects of NO within short intervals of pharmacological testing (∼30–60 min), together with the widespread distribution of putative NOS across multiple non-neuronal cells and tissues, suggest that NO-signaling in ctenophores might have been associated with more systemic functions. For example, assuming that NADPH-d reactivity in *Mnemiopsis* correlates with NOS activity [as in many other preparations (Bredt et al., 1991; Kurenni et al., 1995; Moroz et al., 2000, 2004; Giovine et al., 2001; Bishop et al., 2008; Moroz and Kohn, 2011)] with such broad distribution across cells and tissues, we anticipate potential roles of ctenophore NO-mediated signaling in development, differentiation, morphogenic processes, (neuro)plasticity, immunity, etc. – similar to a plethora of diverse NO functions described in bilaterians, including the vertebrate lineage.

In summary, the studies of NO signaling in ctenophores are in their infancy. Importantly to the field, we know little about NO synthesis, regulation, compartmentalization, and evolution within the group. As critical first steps and future directions, understanding the enzymology of NOS is needed due to its unusual structure. It would be essential to determine the Ca-dependence of ctenophore NOSs, control their expression, and characterize the pharmacology of NOS inhibition, providing tools for future research. Equally important would be the characterization of NOS expression both in development and in adults using *in situ* hybridization and immunohistochemistry, in addition to current observations with NADPH-d histochemistry. Finally, comparative physiological analyses of NO signaling in regulating reproduction, development, feeding, immunity, and complex behavioral integration are desirable.

## Materials and methods

We performed database searches for nitric oxide synthases (NOS) and soluble guanylyl cyclases (sGC) both in animals and non-metazoan organisms. All used NOS and sGC sequences and their sources are presented in Supplementary Table 1.

Protein domains were detected using Pfam (Mistry et al., 2021), UniProt (UniProt Consortium, 2010), SMART (Letunic and Bork, 2018; Letunic et al., 2021), and NCBI Conserved Domain Search (Lu et al., 2020). The reconstruction of 3D structures was based on PDB models using Phyre2 (Kelley et al., 2015), SWISS-MODEL (Waterhouse et al., 2018), and AlphaFold2 (Jumper et al., 2021). Three-dimensional structures were analyzed in PyMol (The PyMol Molecular Graphic System, Version 1.8.6.0 Schrodinger, LLC)^1,2^.

NOS and sGC sequences were aligned with MAFFT v764 88, using the L-INS-i alignment algorithm with 1,000 iterations (Katoh et al., 2005). Phylogenomic ML analyses were performed by IQ-TREE ML ultrafast-bootstrap calculation (Trifinopoulos et al., 2016). The RAxML analyses of NOSs were made by a best-fit LG + F + I + G4 model, and for cyclases, we used a best-fit WAG + F + G4 model. Testing tree branches was done by SH-like aLRT with 1,000 replicates.

We screened publicly available transcriptomic (RNA-seq) datasets of 26 different development stages of *Mnemiopsis* (Levin et al., 2016) for NOS and sGC expression patterns. The original data were presented as TPM (log2(transcript per million (TPM)/100 + 1) and visualized using Pandas, matplotlib libraries in Python.

For analyses of cell-specific expression patters we used reference scRNA-seq data from adult *Mnemiopsis* (Sebé-Pedrós et al., 2018). Specifically, we visually inspected the distribution of genes, UMIs, and % mitochondrial genes across cells choosing a AnnData-file format with a final count matrix of 6,144 cells. We also fileted datasets and tested other criteria for removing cells, but we found that more stringent cutoffs yielded significantly different clustering results and eliminated low-expressed genes from the final visualization (including NOS and sGC-like genes). Standard guidelines for preprocessing, performing principal components analysis, normalization, and clustering were based on Satija et al. (2015).

We ranked differentially expressed genes in each cluster by Wilcoxon rank-sum (Mann–Whitney-U) test and *t*-test (Soneson and Robinson, 2018). As an alternative, we ranked genes using logistic regression (Ntranos et al., 2019). Initial annotation of clusters was based on previously suggested gene markers (Sebé-Pedrós et al., 2018). For visualization of gene expression and other variables, we used dotplots with a dendrogram implemented in Matplotlib, UMAP, and PAGA plottings.

Partition-based graph abstraction (PAGA) connectivity graphs were implemented in ScanPy, Python (Wolf et al., 2019) for mapping the expression for individual genes using Leiden algorithm as a base. Specifically, we employed PAGA to calculate connectivity between merged clusters, retaining all connections. For visualization purposes, we labeled strong edges thicker than others with the threshold 0.03. Finally, we placed the graph nodes on the median UMAP coordinates of the cells in the cluster to preserve the structure of the UMAP embedding and allow direct comparison between the PAGA graph and UMAP plots. To investigate expression changes along different paths in PAGA networks, we calculated diffusion maps and diffusion pseudotime as a proxy of the differentiation distance between all cells.

We used protocols described elsewhere in detail (Kurenni et al., 1995; Moroz, 2000a) for the visualization of NADPH-diaphorase histochemistry known as a marker for NOS activity (Bredt et al., 1991; Moroz, 2000a). Before staining, adult animals (2–5 cm) were fixed in 4% paraformaldehyde in seawater for 45–75 min at room temperature. The staining was also performed at room temperature, in the dark, for 2–5 h in the following solution: β-NADPH sodium salt (1 mM), and Nitro Blue Tetrazolium (0.5 mM) in 0.1 M Tris buffer (pH = 8.0). All reagents were from Sigma). Postfixation, embedding, and visualization were the same as described elsewhere (Moroz, 2000a; Giovine et al., 2001). Considering the highly fragile nature of ctenophore preparations, often dissociated during fixation, we processed different tissue segments separately, using care for all transfers of preparations. The data were obtained from NADPH-d labeling using six animals. The experiments were performed in the spring and summer of 2019–2021.

For behavioral assays, we used freshly collected *Bolinopsis infundibulum*. We used DEA NONOate (Diethylamine NONOate, Abcam, CAS Number 372965-00-9) as a nitric oxide donor. The time series for control behaviors and experimental tests were 30–60 min. We used Fiji/ImageJ and plugins (WRMTRCK, MTrack2) to track and calculate velocities, distances, and tracks. Post-analyses and visualization were done in Python environment (NumPy, Pandas, Matplotlib, SciPy).

## Data availability statement

The datasets presented in this study can be found in online repositories. The names of the repository/repositories and accession number(s) can be found in this article/Supplementary material.

## Author contributions

LM and DR designed the study. KM screened the genome datasets and 37 transcriptomes for the presence of NOS and cyclases. DR and KM was involved in the phylogenetic analyses. DR made 2D/3D protein modeling of NOS and cyclases, performed scRNA-seq and RNA-seq analyses, and involved in the behavioral analyses. LM performed the pharmacological and histochemical tests and wrote the manuscript. DR, KM, and LM confirmed the analyzed data. All authors reviewed and edited the manuscript.

## Footnotes

1
https://www.schrodinger.com/products/pymol

2
https://pymol.org/2/

## Supplementary material

Supplementary Figure 1 Diversification of soluble guanylyl cyclases (sGC) Ctenophora. The catalytic domain (∼190aa) of the soluble guanylate, adenylate, and membrane-type receptor (MR) guanylyl cyclase proteins was used to reconstruct the maximum-likelihood (ML) based phylogenetic tree. Ctenophore soluble cyclases are clustered within three major clades —1/2, 3/4, and NIT-like. Both *Mnemiopsis leidyi* (Ml) and *Ocyropsis crystallina* (Oc) encode five copies of GC proteins, *Pleurobrachia bachei* (Pb) encodes three orthologs comprising one gene product under each cluster. *Pleurobrachia* soluble NIT-like cyclase gene encodes two copies of the catalytic domain shown as a and b. *Mnemiopsis* and *Pleurobrachia* genes are shown in bold font in dark and light blue color. Based on the ML tree membrane receptor type (ANP-like receptor), guanylyl cyclase can be classified under three groups. Both *Pleurobrachia bachei* (Pb) and *Mnemiopsis leidyi* (Ml) encode one gene under these three groups. Similarly, both Pb and Ml encode two genes of the adenylate cyclase family. However, both these species encode two copies of the catalytic domains labeled as a and b. Proteins under each group against all other ctenophore species were retrieved from the transcriptome datasets (Whelan et al., 2015, 2017). The catalytic domain used to build the tree is given in the Supplementary Table 1. Species names are shown in the figure as well as provided in the Supplementary material.

Supplementary Figure 2 Identifying cluster specific genes associated with NO-cGMP signaling in *Mnemiopsis leidyi* based on scRNA-seq data (Sebé-Pedrós et al., 2018) with filtration > 100 genes per cell. This filtration cutoff resulted produced 41 clusters (filtration *n* = 100, 5,461 cells). Without filtration, we produced 24 clusters with 6,144 cells (the main text and figures), and recognized digestive, epithelial, muscle, neuroid-type cells. However, here with the threshold *N* = 100, we lost cells with such low-expressed genes as NOS in tentacles and many cells expressing cyclase genes (see main text).

Supplementary Figure 3 RNA-seq expression profiling of NOS and different cyclases in *Mnemiopsis* development the calculated values are based on Levin et al. (2016), Moreland et al. (2020). Post-fertilization hours are indicated as color dots on the right.

Supplementary Table 1 Sequences of NOS and cyclases used for tree constructions and analyses.

Supplementary Table 2 Sequences of different cyclases identified in comparative ctenophore RNA-seq datasets.

Supplementary Material 1 Alignment of NOSs identified in ctenophores from RNA-seq datasets. All structural domains are marked.

Supplementary Material 2 Alignment of NOSs identified in ctenophores from RNA-seq datasets. All structural domains are marked.
