# Supplementary figures and images for: Nitric oxide signaling in ctenophores
Source: Front Neurosci. 2023 Mar 22;17:1125433. doi: 10.3389/fnins.2023.1125433 (PMC10073611; doi:10.3389/fnins.2023.1125433)

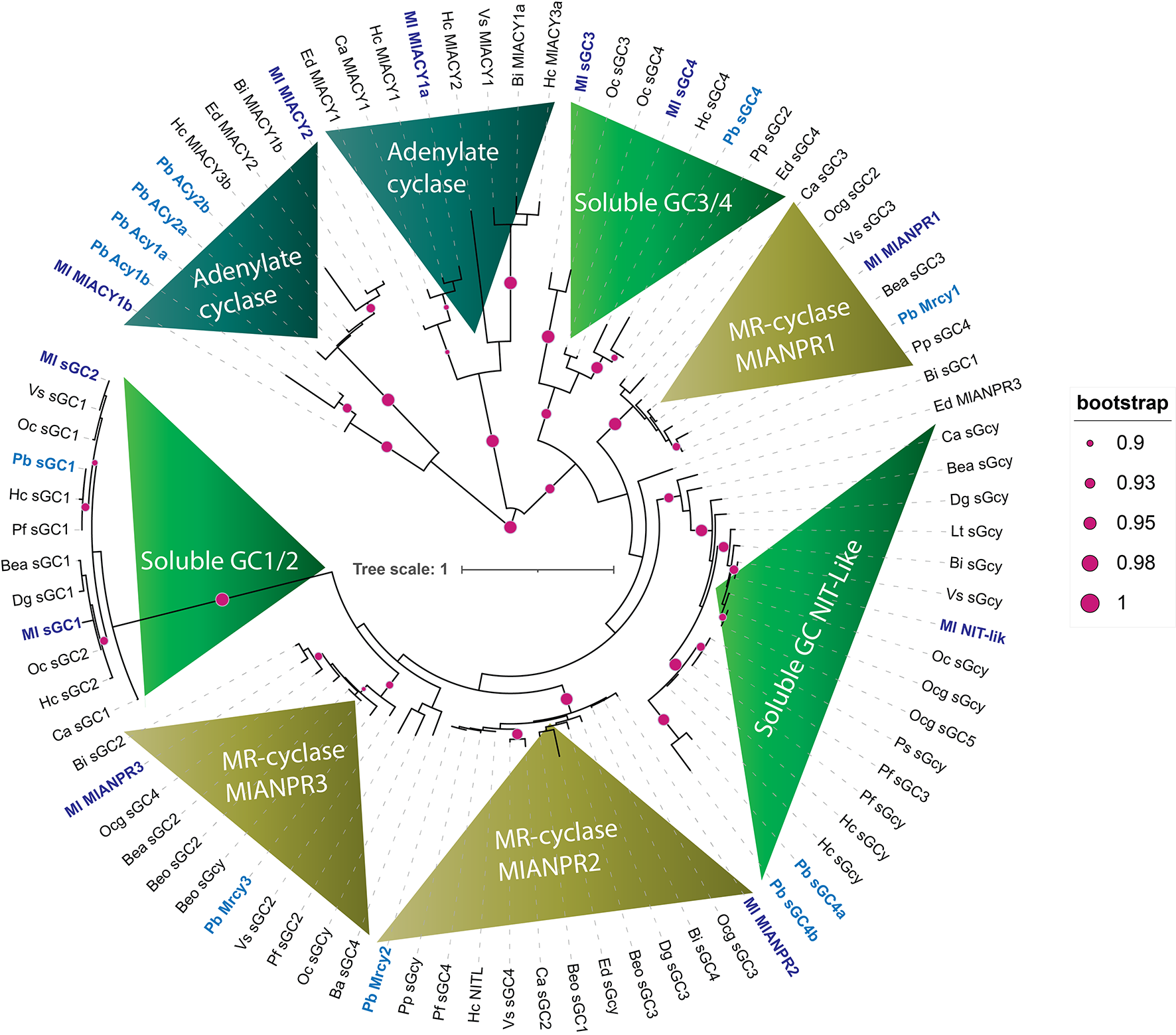

Supplement: Supplementary Figure 1 — Diversification of soluble guanylyl cyclases (sGC) Ctenophora. The catalytic domain (∼190aa) of the soluble guanylate, adenylate, and membrane-type receptor (MR) guanylyl cyclase proteins was used to reconstruct the maximum-likelihood (ML) based phylogenetic tree. Ctenophore soluble cyclases are clustered within three major clades —1/2, 3/4, and NIT-like. Both Mnemiopsis leidyi (Ml) and Ocyropsis crystallina (Oc) encode five copies of GC proteins, Pleurobrachia bachei (Pb) encodes three orthologs comprising one gene product under each cluster. Pleurobrachia soluble NIT-like cyclase gene encodes two copies of the catalytic domain shown as a and b. Mnemiopsis and Pleurobrachia genes are shown in bold font in dark and light blue color. Based on the ML tree membrane receptor type (ANP-like receptor), guanylyl cyclase can be classified under three groups. Both Pleurobrachia bachei (Pb) and Mnemiopsis leidyi (Ml) encode one gene under these three groups. Similarly, both Pb and Ml encode two genes of the adenylate cyclase family. However, both these species encode two copies of the catalytic domains labeled as a and b. Proteins under each group against all other ctenophore species were retrieved from the transcriptome datasets (Whelan et al., 2015, 2017). The catalytic domain used to build the tree is given in the Supplementary Table 1. Species names are shown in the figure as well as provided in the Supplementary material. [file Image_1.TIF]

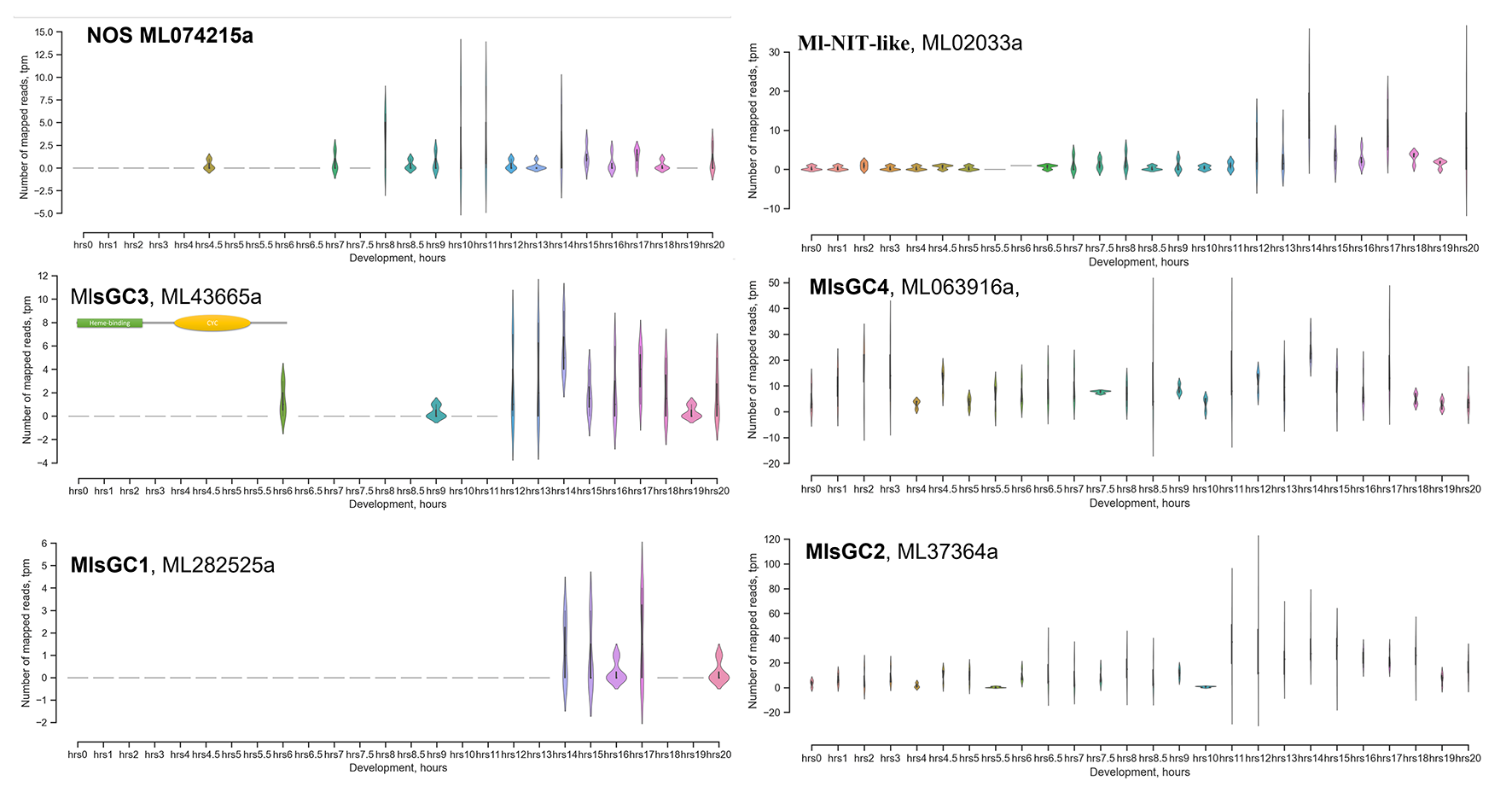

Supplement: Supplementary Figure 3 — RNA-seq expression profiling of NOS and different cyclases in Mnemiopsis development the calculated values are based on Levin et al. (2016), Moreland et al. (2020). Post-fertilization hours are indicated as color dots on the right. [file Image_3.TIF]
