# Supplementary material for: Nitric oxide signaling in ctenophores
Source: Front Neurosci. 2023 Mar 22;17:1125433. doi: 10.3389/fnins.2023.1125433 (PMC10073611; doi:10.3389/fnins.2023.1125433)
Supplement: Supplementary Material 1 — Alignment of NOSs identified in ctenophores from RNA-seq datasets. All structural domains are marked. [file Data_Sheet_1.PDF]

```
                                                        PDZ
Coeloplana_astericola_NOS      ............................................................
Euplokamis_dunlapae_NOS        ............................................................
Bolinopsis_infundibulum_NOS    ............................................................
Ocyropsis_crystallina_NOS      ............................................................
Lobatolampea_tetragona_NOS     ............................................................
Homo_sapiens_NOS2              ............................................................
Homo_sapiens_NOS1              MEDHMFGVQQIQPNVISVRLFKRKVGGLGFLVKERVSKPPVIISDLIRGGAAEQSGLIQA
Homo_sapiens_NOS3              ............................................................

Coeloplana_astericola_NOS      ............................................................
Euplokamis_dunlapae_NOS        ............................................................
Bolinopsis_infundibulum_NOS    ............................................................
Ocyropsis_crystallina_NOS      ............................................................
Lobatolampea_tetragona_NOS     ............................................................
Homo_sapiens_NOS2              .....................................................MACPWKF
Homo_sapiens_NOS1              GDIILAVNGRPLVDLSYDSALEVLRGIASETHVVLILRGPEGFTTHLETTFTGDGTPKTI
Homo_sapiens_NOS3              .....................................................MGNLKS.

Coeloplana_astericola_NOS      ............................................................
Euplokamis_dunlapae_NOS        ............................................................
Bolinopsis_infundibulum_NOS    ............................................................
Ocyropsis_crystallina_NOS      ............................................................
Lobatolampea_tetragona_NOS     ............................................................
Homo_sapiens_NOS2              LFKTKF....HQYAMNGEKDINNNVEKAPCATSSPVTQDDLQYHN...............
Homo_sapiens_NOS1              RVTQPLGPPTKAVDLSHQPPAGKEQPLAVDGASGPGNGPQHAYDDGQEAGSLPHANGLAP
Homo_sapiens_NOS3              .VAQEPGPPCG.........LGLGLGLGLCGKQGPAT.......................

Coeloplana_astericola_NOS      ............................................................
Euplokamis_dunlapae_NOS        ............................................................
Bolinopsis_infundibulum_NOS    ............................................................
Ocyropsis_crystallina_NOS      ............................................................
Lobatolampea_tetragona_NOS     ............................................................
Homo_sapiens_NOS2              ..LSKQQNE...................................................
Homo_sapiens_NOS1              RPPGQDPAKKATRVSLQGRGENNELLKEIEPVLSLLTSGSRGVKGGAPAKAEMKDMGIQV
Homo_sapiens_NOS3              ..PAPEPSR...................................................

Coeloplana_astericola_NOS      ............................................................
Euplokamis_dunlapae_NOS        ............................................................
Bolinopsis_infundibulum_NOS    ............................................................
Ocyropsis_crystallina_NOS      ............................................................
Lobatolampea_tetragona_NOS     ............................................................
Homo_sapiens_NOS2              ..............................SPQPLVE..TGKKSPESLVKL....DATPL
Homo_sapiens_NOS1              DRDLDGKSHKPLPLGVENDRVFNDLWGKGNVPVVLNNPYSEKEQPPTSGKQSPTKNGSPS
Homo_sapiens_NOS3              ..............................APASLLPPAPEHSPPSSPLTQ.......PP

                                         HPL                Zn       Pterin
Coeloplana_astericola_NOS      ............................................................
Euplokamis_dunlapae_NOS        ............................................................
Bolinopsis_infundibulum_NOS    ............................................................
Ocyropsis_crystallina_NOS      ............................................................
Lobatolampea_tetragona_NOS     ............................................................
Homo_sapiens_NOS2              SSPRHVRIKNWGSGMTFQDTLHHKAKGILTCRSKSCLGSIMTPKSLTRGPRDKPTPPDEL
Homo_sapiens_NOS1              KCPRFLKVKNWETEVVLTDTLHLKSTLETGCTEYICMGSIMHPSQHARRPE.DVRTKGQL
Homo_sapiens_NOS3              EGPKFPRVKNWEVGSITYDTLSAQAQQDGPCTPRRCLGSLVFPRKLQGRPSPGPPAPEQL

                                                                                    HEME
Coeloplana_astericola_NOS      ............................................................
Euplokamis_dunlapae_NOS        ............................................................
Bolinopsis_infundibulum_NOS    ............................................................
Ocyropsis_crystallina_NOS      ............................................................
Lobatolampea_tetragona_NOS     ...MEEFIENYYNDCKHSKPLMSKEDRLTEIYLQLETEGDYFHTEDELVWAAKTAWRNAS
Homo_sapiens_NOS2              LPQAIEFVNQYYGSFKEAK.IEEHLARVEAVTKEIETTGTYQLTGDELIFATKQAWRNAP
Homo_sapiens_NOS1              FPLAKEFIDQYYSSIKRFG.SKAHMERLEEVNKEIDTTSTYQLKDTELIYGAKHAWRNAS
Homo_sapiens_NOS3              LSQARDFINQYYSSIKRSG.SQAHEQRLQEVEAEVAATGTYQLRESELVFGAKQAWRNAP
```

```
Coeloplana_astericola_NOS      ............................................................
Euplokamis_dunlapae_NOS        ............................................................
Bolinopsis_infundibulum_NOS    ............................................................
Ocyropsis_crystallina_NOS      ............................................................
Lobatolampea_tetragona_NOS     RCSGRNLWQTLKVIDKRDAKTGPEIFEAVCT....................KDPNSPGVR
Homo_sapiens_NOS2              RCIGRIQWSNLQVFDARSCSTAREMFEHICRHVRYSTNNGNIRSAITVFPQRSDGKHDFR
Homo_sapiens_NOS1              RCVGRIQWSKLQVFDARDCTTAHGMFNYICNHVKYATNKGNLRSAITIFPQRTDGKHDFR
Homo_sapiens_NOS3              RCVGRIQWGKLQVFDARDCRSAQEMFTYICNHIKYATNRGNLRSAITVFPQRCPGRGDFR
```

PF02898: NO_synthase

```
Coeloplana_astericola_NOS      ............................................................
Euplokamis_dunlapae_NOS        ..........................................MFDLLPVIITDADQSTEM
Bolinopsis_infundibulum_NOS    ............................................................
Ocyropsis_crystallina_NOS      ............................................................
Lobatolampea_tetragona_NOS     IWNSLILSFAGHVKPNGETIGDPKNVFLTNVAKTYGWKPKCEPFELLPLIITDWYQKTAI
Homo_sapiens_NOS2              VWNAQLIRYAGYQMPDGSIRGDPANVEFTQLCIDLGWKPKYGRFDVVPLVLQANGRDPEL
Homo_sapiens_NOS1              VWNSQLIRYAGYKQPDGSTLGDPANVQFTEICIQQGWKPPRGRFDVLPLLLQANGNDPEL
Homo_sapiens_NOS3              IWNSQLVRYAGYRQQDGSVRGDPANVEITELCIQHGWTPGNGRFDVLPLLLQAPDDPPEL
```

Cav

```
Coeloplana_astericola_NOS      .....DLKSYVIDIKHSSIPAISKLGIKWYAIPSVSFMLLEAGGIQYTGAPIAGFYQDTE
Euplokamis_dunlapae_NOS        FELPNDIQGYTVDIFHPKIPAIANLGLKWYAIPSVTSMMLEAGGIQYTCAPITGFFQDSE
Bolinopsis_infundibulum_NOS    ............................................................
Ocyropsis_crystallina_NOS      ...................................SSMMLEAGGIQYTCCHIAGFFQDTE
Lobatolampea_tetragona_NOS     FELPDDIKNYLVDISDPKYPELSELKLKWYAIPAVSAMMLEAGGIQYTCTPIAGFFQDTE
Homo_sapiens_NOS2              FEIPPDLV.LEVAMEHPKYEWFRELELKWYALPAVANMLLEVGGLEFPGCPFNGWYMGTE
Homo_sapiens_NOS1              FQIPPELV.LEVPIRHPKFEWFKDLGLKWYGLPAVSNMLLEIGGLEFSACPFSGWYMGTE
Homo_sapiens_NOS3              FLLPPELV.LEVPLEHPTLEWFAALGLRWYALPAVSNMLLEIGGLEFPAAPFSGWYMSTE
```

PF02898: NO_synthase

```
Coeloplana_astericola_NOS      VSIMDLLARSRYNLLETV..................VGLEITRAVYESYKNKGVTIV...
Euplokamis_dunlapae_NOS        VSVLDLLASPRYNLLEPVGRVMELDVTSNLSYWKADVCTELTKAVNCSYREAGVTIIDHF
Bolinopsis_infundibulum_NOS    ...MNLLAESRYNLMEPINRAIKLDVSKNSTYWKCTVATELTRAVYHSFKLARVSMT...
Ocyropsis_crystallina_NOS      ISVMNMLGQGRYNLMEPIGRALKLDVSMNATYWKCRVTIELTTAVYYSFKKA........
Lobatolampea_tetragona_NOS     VSVLDLLAESRYNLMEPIGRLLGLDVGSNTTYWKCDVATILTRAVYHSYKKAGVTMI...
Homo_sapiens_NOS2              IGVRDFCDVQRYNILEEVGRRMGLETHKLASLWKDQAVVEINIAVLHSFQKQNVTIM...
Homo_sapiens_NOS1              IGVRDYCDNSRYNILEEVAKKMNLDMRKTSSLWKDQALVEINIAVLYSFQSDKVTIV...
Homo_sapiens_NOS3              IGTRNLCDPHRYNILEDVAVCMDLDTRTTSSLWKDKAAVEINVAVLHSYQLAKVTIV...
```

```
Coeloplana_astericola_NOS      ............DHFTISERFQSFMKEELELRGGCPADWVWIVPPLSSGVVPTFHQEMLR
Euplokamis_dunlapae_NOS        TYNMGAYNEVYLDHFTMSEEFHTFMAEEVKTRGGCPADWLWVVPAMSSGITPVFHQEMAR
Bolinopsis_infundibulum_NOS    ............DHFTLSVSFHLFMKEEMRTRGGCPTDWLWVVPPMSGGLVPTFHQEMLR
Ocyropsis_crystallina_NOS      ............................................................
Lobatolampea_tetragona_NOS     ............DHFSQADKFVEHMHEEISNRGGCPADWVWVVPSLSSGLVPTYHQEMLR
Homo_sapiens_NOS2              ............DHHSAAESFMKYMQNEYRSRGGCPADWIWLVPPMSGSITPVFHQEMLN
Homo_sapiens_NOS1              ............DHHSATESFIKHMENEYRCRGGCPADWVWIVPPMSGSITPVFHQEMLN
Homo_sapiens_NOS3              ............DHHAATASFMKHLENEQKARGGCPADWAWIVPPISGSLTPVFHQEMVN
```

CaM

```
Coeloplana_astericola_NOS      YTLHPSYEYEIDAEEYFVKPRKK........LTFRGVAWIVYFFVMKAVEKFRQRKMMSI
Euplokamis_dunlapae_NOS        YMMSPSYEYQSSAEEFFKKKKERR.......ASFYSVGWTVLCFVSMLRKRFKERKKIAF
Bolinopsis_infundibulum_NOS    YTLSPSYEYQPGPEMFFRKRRNK........VSFRALATTVMKCLEMMREP.........
Ocyropsis_crystallina_NOS      ............................................................
Lobatolampea_tetragona_NOS     YALSPSYEYQSEPEMFFRREKIR.....................................
Homo_sapiens_NOS2              YVLSPFYYYQVEAWKTHVWQDEKRRPK.RREIPLKVLVKAVLFACMLMRKTMASRVRVTI
Homo_sapiens_NOS1              YRLTPSFEYQPDPWNTHVWKGTNGTPTKRRAIGFKKLAEAVKFSAKLMGQAMAKRVKATI
Homo_sapiens_NOS3              YFLSPAFRYQPDPWKGSAAKGTGITRK....KTFKEVANAVKISASLMGTVMAKRVKATI
```

PF00258: Flavodoxin_1

```
Coeloplana_astericola_NOS      VYATETGTSREFAKTTSELFEYGFNVNLMSMDEVDQDETEFYQKIKDSVFTIFIVSTFGV
Euplokamis_dunlapae_NOS        IYATETGTAFN.................................................
Bolinopsis_infundibulum_NOS    .....................................................IASTFGE
Ocyropsis_crystallina_NOS      ............................................................
Lobatolampea_tetragona_NOS     .....NGSSHKFAKNAEQTFAAYFKIITIRLDEIKCIE.DFVSRAESSFLSLVITSTFGC
Homo_sapiens_NOS2              LFATETGKSEALAWDLGALFSCAFNPKVVCMDK......YRLSCLEEERLLLVVTSTFGN
Homo_sapiens_NOS1              LYATETGKSQAYAKTLCEIFKHAFDAKVMSMEE......YDIVHLEHETLVLVVTSTFGN
Homo_sapiens_NOS3              LYGSETGRAQSYAQQLGRLFRKAFDPRVLCMDE......YDVVSLEHETLVLVVTSTFGN
```

AIL

```
Coeloplana_astericola_NOS      GESPLMAATFAKQLKDLE........................................NY
Euplokamis_dunlapae_NOS        ............................................................
Bolinopsis_infundibulum_NOS    GGPPSMAEEFHRNLLNKS..........................................
Ocyropsis_crystallina_NOS      ............................................................
Lobatolampea_tetragona_NOS     GGPPTMAIKFKESMENFI..........................................
Homo_sapiens_NOS2              GDCPGNGEKLKKSLFMLK..........................................
Homo_sapiens_NOS1              GDPPENGEKFGCALMEMRHP...NSVQEERKSYKVRFNSVSSYSDSQKSSGDGPDLRDNF
Homo_sapiens_NOS3              GDPPENGESFAAALMEMSGPYNSSPRPEQHKSYKIRFNSISCSDPLVSSWRRKRKESSNT
```

FMN

```
Coeloplana_astericola_NOS      KGVKWFEDIKYGVFGLGSSDY.PNLAAFGKTIHNRLQDVGGEPLVDVGIGDDQKDQLDAF
Euplokamis_dunlapae_NOS        ...........................................................F
Bolinopsis_infundibulum_NOS    ...YRFDGMKYAVFGLGSSQYIETFANFGKTLDREMEIGGGTRITEVGFGDDQGNQKLEF
Ocyropsis_crystallina_NOS      ............................................................
Lobatolampea_tetragona_NOS     .....................................SLGGNEVAEIGFGDDQADQMAAF
Homo_sapiens_NOS2              ...ELNNKFRYAVFGLGSSMY.PRFCAFAHDIDQKLSHLGASQLTPMGEGDELSGQEDAF
Homo_sapiens_NOS1              ESAGPLANVRFSVFGLGSRAY.PHFCAFGHAVDTLLEELGGERILKMREGDELCGQEEAF
Homo_sapiens_NOS3              DSAGALGTLRFCVFGLGSRAY.PHFCAFARAVDTRLEELGGERLLQLGQGDELCGQEEAF
```

```
Coeloplana_astericola_NOS      EQWINKMYQVSCGRFL.....PSAADGTGSSLNTLYRWRLENPRPLKEVLQAHVGSG...
Euplokamis_dunlapae_NOS        TQFAITLFQKAFDTAT............................................
Bolinopsis_infundibulum_NOS    DNWIKALYEKCCKDYLPLSGRPQGTAIEGATLDKVYRWRYSDKRSLSECFQEEIGPS...
Ocyropsis_crystallina_NOS      ............................................................
Lobatolampea_tetragona_NOS     KVWTNNILDWCFRTSS.....IDRFTTGTQTRQAVYRWQYVDKVSVLEAHQSHALAS...
Homo_sapiens_NOS2              RSWAVQTFKAACETFD...VRGKQHIQIPKLYTSNVTWDPHHYRLVQDSQPLDLSKALSS
Homo_sapiens_NOS1              RTWAKKVFKAACDVFC.VGDDVNIEKANNSLISNDRSWKRNKFRLTFVAEAPELTQGLSN
Homo_sapiens_NOS3              RGWAQAAFQAACETFC...VGEDAKAAARDIFSPKRSWKRQRYRLSAQAEGLQLLPGLIH
```

FAD-PPi

```
Coeloplana_astericola_NOS      ...GHVKSFTVTGRSVLSLDKQDERYLLIQFSSDPD.DESLLEYKPGDHLAILPRNSDDL
Euplokamis_dunlapae_NOS        ............................................................
Bolinopsis_infundibulum_NOS    ...EKMFEFTVKAKKSLSPDGASEKYYLLTLSYNADPKRDMLLFLPGQHIGIFPKNLSSV
Ocyropsis_crystallina_NOS      ............................................................
Lobatolampea_tetragona_NOS     ............................................................
Homo_sapiens_NOS2              MHAKNVFTMRLKSRQNLQSPTSSRATILVELSCE...DGQGLNYLPGEHLGVCPGNQPAL
Homo_sapiens_NOS1              VHKKRVSAARLLSRQNLQSPKSSRSTIFVRLHTN...GSQELQYQPGDHLGVFPGNHEDL
Homo_sapiens_NOS3              VHRRKMFQATIRSVENLQSSKSTRATILVRLDTG...GQEGLQYQPGDHIGVCPPNRPGL
```

```
Coeloplana_astericola_NOS      VNFVAGNLTNQPFDDLPLKLEVKGAGSN......TWNLSSYPNGMNYRQFLSYLVDLHHI
Euplokamis_dunlapae_NOS        ............................................................
Bolinopsis_infundibulum_NOS    KEKIVRRLSDVPFSSIPLIIE...EKIAIHEPWRQW..NPNYSGLTIDEFFGNVADLKQI
Ocyropsis_crystallina_NOS      ............................................................
Lobatolampea_tetragona_NOS     ............................................................
Homo_sapiens_NOS2              VQGILERVVDGPTPHQTVRLEALDESGS......YWVSDKRLPPCSLSQALTYFLDITTP
Homo_sapiens_NOS1              VNALIERLEDAPPVNQMVKVELLEERNTALGVISNWTDELRLPPCTIFQAFKYYLDITTP
Homo_sapiens_NOS3              VEALLSRVEDPPAPTEPVAVEQL.EKGSPGGPPPGWVRDPRLPPCTLRQALTFFLDITSP
```

PF00667: FAD_binding_1

```
Coeloplana_astericola_NOS      PMDK..VLAYINTREFIHPLDHDMDIKAE............TCDLDDVLEAVRPNKLESP
Euplokamis_dunlapae_NOS        ............................................................
Bolinopsis_infundibulum_NOS    PANLHETLEKDDKNGENGHKTSREDQLEE...........................LDSA
Ocyropsis_crystallina_NOS      ............................................................
Lobatolampea_tetragona_NOS     ............................................................
Homo_sapiens_NOS2              PTQL..LLQKLAQVATEEPERQRLEALCQ.PSEYSKWKFTNSPTFLEVLEEFPSLRVSAG
Homo_sapiens_NOS1              PTPL..QLQQFASLATSEKEKQRLLVLSKGLQEYEEWKWGKNPTIVEVLEEFPSIQMPAT
Homo_sapiens_NOS3              PSPQ..LLRLLSTLAEEPREQQELEALSQDPRRYEEWKWFRCPTLLEVLEQFPSVALPAP
```

FAD-Iso

```
Coeloplana_astericola_NOS      GLLARLDEMDKRLYSVASS....PLEHGKVSILISITEYLKNDKVKEGLCSSYLKNLKLG
Euplokamis_dunlapae_NOS        ............................................................
Bolinopsis_infundibulum_NOS    KFIGQIPTIKRRLFSIASC....QNDSHTLNILIALHEFMSDGKIVGGLTSDFVRQAPLG
Ocyropsis_crystallina_NOS      ............................................................
Lobatolampea_tetragona_NOS     ............................................................
Homo_sapiens_NOS2              FLLSQLPILKPRFYSISSSRDHTPTEIHLTVAVVTYHTRDGQGPLHHGVCSTWLNSLKPQ
Homo_sapiens_NOS1              LLLTQLSLLQPRYYSISSSPDMYPDEVHLTVAIVSYRTRDGEGPIHHGVCSSWLNRIQAD
Homo_sapiens_NOS3              LLLTQLPLLQPRYYSVSSAPSTHPGEIHLTVAVLAYRTQDGLGPLHYGVCSTWLSQLKPG
```

NADPH-Ribose

```
Coeloplana_astericola_NOS      DTVYGYYISTNFQMRLDEDPTKPMILVSAGSGYAPFRSFIKHREVQSKC.GIRTGKILVL
Euplokamis_dunlapae_NOS        ............................................................
Bolinopsis_infundibulum_NOS    EKIQGYLSKGDEQMRLPQNPRLPLLLVSVGSGFAPFMSFIEARERAART.GIKTGPIFIF
Ocyropsis_crystallina_NOS      ............................................................
Lobatolampea_tetragona_NOS     ............................................................
Homo_sapiens_NOS2              DPVPCF.VRNASGFHLPEDPSHPCILIGPGTGIAPFRSFWQQRLHDSQHKGVRGGRMTLV
Homo_sapiens_NOS1              ELVPCF.VRGAPSFHLPRNPQVPCILVGPGTGIAPFRSFWQQRQFDIQHKGMNPCPMVLV
Homo_sapiens_NOS3              DPVPCF.IRGAPSFRLPPDPSLPCILVGPGTGIAPFRGFWQERLHDIESKGLQPTPMTLV
```

PF00175: NAD_binding_1

```
Coeloplana_astericola_NOS      FGCRNKNVDILKDETDSLTAKKILNIERHTAFSREAGIQKK.......YVQDLVLEH.ST
Euplokamis_dunlapae_NOS        ............................................................
Bolinopsis_infundibulum_NOS    HGCRYKEHDFL..DSLLESAATVLNIKTFRAYSRSEPDCKKPDQNGRGYIQDLIAEQ..G
Ocyropsis_crystallina_NOS      ............................................................
Lobatolampea_tetragona_NOS     ...........................................................K
Homo_sapiens_NOS2              FGCRRPDEDHIYQEEMLEMAQKGVLHAVHTAYSRLPGKPKV.......YVQDILRQQLAS
Homo_sapiens_NOS1              FGCRQSKIDHIYREETLQAKNKGVFRELYTAYSREPDKPKK.......YVQDILQEQLAE
Homo_sapiens_NOS3              FGCRCSQLDHLYRDEVQNAQQRGVFGRVLTAFSREPDNPKT.......YVQDILRTELAA
```

NADPH-A　　　　NADPH

```
Coeloplana_astericola_NOS      EIENMLLKQDGTIYVCGGQKMAIGVQENVESILAKSGSNVTVD.......MLKKKK....
Euplokamis_dunlapae_NOS        ............................................................
Bolinopsis_infundibulum_NOS    EVVARVSRDGGYMYSCGGSEAVAGVRGQLEKTLLEQGCVSLQE.......MMCRKRYQEE
Ocyropsis_crystallina_NOS      ............................................................
Lobatolampea_tetragona_NOS     NIVKLLCPKQNYLIN.............................................
Homo_sapiens_NOS2              EVLRVLHKEPGHLYVCGDVRMARDVAHTLKQLVAAKLKLNEEQVEDYFFQLKSQKRYHED
Homo_sapiens_NOS1              SVYRALKEQGGHIYVCGDVTMAADVLKAIQRIMTQQGKLSAEDAGVFISRMRDDNRYHED
Homo_sapiens_NOS3              EVHRVLCLERGHMFVCGDVTMATNVLQTVQRILATEGDMELDEAGDVIGVLRDQQRYHED
```

```
Coeloplana_astericola_NOS      .............................................
Euplokamis_dunlapae_NOS        .............................................
Bolinopsis_infundibulum_NOS    KFG..........................................
Ocyropsis_crystallina_NOS      .............................................
Lobatolampea_tetragona_NOS     .............................................
Homo_sapiens_NOS2              IFGAVFPYEAKKDRVAVQPSSLEMSAL..................
Homo_sapiens_NOS1              IFGVTLRTYEVTNRLRSESIAFIEESKKDTDEVFSS.........
Homo_sapiens_NOS3              IFGLTLRTQEVTSRIRTQSFSLQERQLRGAVPWAFDPPGSDTNSP
```
