# Supplementary material for: Nitric oxide signaling in ctenophores
Source: Front Neurosci. 2023 Mar 22;17:1125433. doi: 10.3389/fnins.2023.1125433 (PMC10073611; doi:10.3389/fnins.2023.1125433)
Supplement: Supplementary Material 2 — Alignment of NOSs identified in ctenophores from RNA-seq datasets. All structural domains are marked. [file Data_Sheet_2.PDF]

```
Hs_SGCY1     ............................................................
Ml_sGC3      ............................................................
Oc_sGC3      ............................................................
Ml_sGC1      ............................................................
Ca_sGC1      ............................................................
Pf_sGC2      ............................................................
Pb_sGC2      ............................................................
Oc_sGC2      ............................................................
Ml_sGC2      ............................................................
Vs_sGC2      ............................................................
Hc_sGC1      ............................................................
Dg_sGC1      ............................................................
Bea_sGC1     ............................................................
Hc_sGC2      ............................................................
Oc_sGC1      ............................................................
Ml_sGC4      ............................................................
Oc_sGC4      ............................................................
Hc_sGC4      ............................................................
Pp_sGC2      ............................................................
Pb_sGC4      ............................................................
Hs_SGCY3     ............................................................
Hs_SGCY2     ............................................................
Ed_sGC4      ............................................................
Pb_Nitl      ............................................................
Ed_Nitl      ............................................................
Hc_Nitl      MMMWPFLLLTSSLFYIVHSGAPIYGFMDLFRPDFEQQQGALWSENPSFLGFEVGPGALVA
Pf_Nitl2     ............................................................
Pf_Nitl      ............................................................
Ps_Nitl      ............................................................
Ca_Nitl      ............................................................
Bea_Nitl     ............................................................
Bi_sGC1      ............................................................
Bi_Nitl      ............................................................
Dg_Nitl      ............................................................
Lt_Nitl      ............................................................
Ml_NIT-like  ............................................................
Vs_Nitl      ............................................................
Oc_Nitl      ............................................................
Ocg_Nitl     ............................................................
Ocg_Nitl2    ............................................................
```

```
Hs_SGCY1     ............................................................
Ml_sGC3      ............................................................
Oc_sGC3      ............................................................
Ml_sGC1      ............................................................
Ca_sGC1      ............................................................
Pf_sGC2      ............................................................
Pb_sGC2      .................................MLGELLFNQLTCPYTKAGQCKLAYLKF
Oc_sGC2      ............................................................
Ml_sGC2      ............................................................
Vs_sGC2      ............................................................
Hc_sGC1      ............................................................
Dg_sGC1      ............................................................
Bea_sGC1     ............................................................
Hc_sGC2      ............................................................
Oc_sGC1      ............................................................
Ml_sGC4      ............................................................
Oc_sGC4      ............................................................
Hc_sGC4      ............................................................
Pp_sGC2      ............................................................
Pb_sGC4      ............................................................
Hs_SGCY3     ............................................................
Hs_SGCY2     ............................................................
Ed_sGC4      ............................................................
Pb_Nitl      ............................................................
Ed_Nitl      ............................................................
Hc_Nitl      LDELGKKNARGDLELLTAWDIERTEWDKAQTRKNLSTIIECGDEWKTERCLENLRSTGRQ
Pf_Nitl2     ............................................................
Pf_Nitl      ............................................................
Ps_Nitl      ............................................................
Ca_Nitl      ............................................................
Bea_Nitl     ............................................................
Bi_sGC1      ............................................................
Bi_Nitl      ............................................................
Dg_Nitl      ............................................................
Lt_Nitl      ............................................................
Ml_NIT-like  ............................................................
Vs_Nitl      ............................................................
Oc_Nitl      ............................................................
Ocg_Nitl     ............................................................
Ocg_Nitl2    ............................................................
```

```
Hs_SGCY1      ............................................................
Ml_sGC3       ............................................................
Oc_sGC3       ............................................................
Ml_sGC1       ............................................................
Ca_sGC1       ............................................................
Pf_sGC2       ............................................................
Pb_sGC2       KECVSKTQGLFLSSAKTQNAQKANLYYLQQTCLILIRSTLTLKQASFRIANLMRVLCSEA
Oc_sGC2       ............................................................
Ml_sGC2       ............................................................
Vs_sGC2       ............................................................
Hc_sGC1       ............................................................
Dg_sGC1       ............................................................
Bea_sGC1      ............................................................
Hc_sGC2       ............................................................
Oc_sGC1       ............................................................
Ml_sGC4       ............................................................
Oc_sGC4       ............................................................
Hc_sGC4       ............................................................
Pp_sGC2       ............................................................
Pb_sGC4       ............................................................
Hs_SGCY3      ............................................................
Hs_SGCY2      ............................................................
Ed_sGC4       ............................................................
Pb_Nitl       ............................................................
Ed_Nitl       ............................................................
Hc_Nitl       PTLIIGSHKDLASQTSSLHSFINSGLFVYCSADISNSAVVSGPTKDSKGLLNTVRMDNSV
Pf_Nitl2      ............................................................
Pf_Nitl       ............................................................
Ps_Nitl       ............................................................
Ca_Nitl       ............................................................
Bea_Nitl      ............................................................
Bi_sGC1       ............................................................
Bi_Nitl       ............................................................
Dg_Nitl       ............................................................
Lt_Nitl       ............................................................
Ml_NIT-like   ............................................................
Vs_Nitl       ............................................................
Oc_Nitl       ............................................................
Ocg_Nitl      ............................................................
Ocg_Nitl2     ............................................................
```

```
Hs_SGCY1      ............................................................
Ml_sGC3       ............................................................
Oc_sGC3       ............................................................
Ml_sGC1       ............................................................
Ca_sGC1       ............................................................
Pf_sGC2       ............................................................
Pb_sGC2       VQDPTKVEANVRAQAAERQKKHLKSNAERQLTKEQKTEKKIAKLTEDTTDGVHVTVFKID
Oc_sGC2       ............................................................
Ml_sGC2       ............................................................
Vs_sGC2       ............................................................
Hc_sGC1       ............................................................
Dg_sGC1       ............................................................
Bea_sGC1      ............................................................
Hc_sGC2       ............................................................
Oc_sGC1       ............................................................
Ml_sGC4       ............................................................
Oc_sGC4       ............................................................
Hc_sGC4       ............................................................
Pp_sGC2       ............................................................
Pb_sGC4       ............................................................
Hs_SGCY3      ............................................................
Hs_SGCY2      ............................................................
Ed_sGC4       ............................................................
Pb_Nitl       ............................................................
Ed_Nitl       ............................................................
Hc_Nitl       FSGNVQAFLKHYGWTQHLFIISGVFQADYRSGAVWDDDTLNLVDTSSQKKRVRFYPFSFM
Pf_Nitl2      ............................................................
Pf_Nitl       ............................................................
Ps_Nitl       ............................................................
Ca_Nitl       ............................................................
Bea_Nitl      ............................................................
Bi_sGC1       ............................................................
Bi_Nitl       ............................................................
Dg_Nitl       ............................................................
Lt_Nitl       ............................................................
Ml_NIT-like   ............................................................
Vs_Nitl       ............................................................
Oc_Nitl       ............................................................
Ocg_Nitl      ............................................................
Ocg_Nitl2     ............................................................
```

```
Hs_SGCY1      ............................................................
Ml_sGC3       ............................................................
Oc_sGC3       ............................................................
Ml_sGC1       ............................................................
Ca_sGC1       ............................................................
Pf_sGC2       ............................................................
Pb_sGC2       NLSSPQHKYKVETNAQQFLLTGIMLMHKDHNLVIVEGGPKALRKFKRLMLLRIRWNEDVR
Oc_sGC2       ............................................................
Ml_sGC2       ............................................................
Vs_sGC2       ............................................................
Hc_sGC1       ............................................................
Dg_sGC1       ............................................................
Bea_sGC1      ............................................................
Hc_sGC2       ............................................................
Oc_sGC1       ............................................................
Ml_sGC4       ............................................................
Oc_sGC4       ............................................................
Hc_sGC4       ............................................................
Pp_sGC2       ............................................................
Pb_sGC4       ............................................................
Hs_SGCY3      ............................................................
Hs_SGCY2      ............................................................
Ed_sGC4       ............................................................
Pb_Nitl       ............................................................
Ed_Nitl       ............................................................
Hc_Nitl       EIKDGNTDPFREDARKERTELYEEIFKGTAIKHHSRIVLLFGSQRMVRDMMLEAYDAGMM
Pf_Nitl2      ............................................................
Pf_Nitl       ............................................................
Ps_Nitl       ............................................................
Ca_Nitl       ..........................................................MA
Bea_Nitl      ............................................................
Bi_sGC1       ............................................................
Bi_Nitl       ............................................................
Dg_Nitl       ............................................................
Lt_Nitl       ............................................................
Ml_NIT-like   ............................................................
Vs_Nitl       ............................................................
Oc_Nitl       ............................................................
Ocg_Nitl      ............................................................
Ocg_Nitl2     ............................................................
```

```
Hs_SGCY1      ............................................................
Ml_sGC3       ............................................................
Oc_sGC3       ............................................................
Ml_sGC1       ............................................................
Ca_sGC1       ............................................................
Pf_sGC2       ............................................................
Pb_sGC2       NRKRCKTLRMHGQQYTNFTFLAGYKKDTLLFKVDIKINCRRDFCRKGLSRQIQLSKSLGL
Oc_sGC2       ............................................................
Ml_sGC2       ............................................................
Vs_sGC2       ............................................................
Hc_sGC1       ............................................................
Dg_sGC1       ............................................................
Bea_sGC1      ............................................................
Hc_sGC2       ............................................................
Oc_sGC1       ............................................................
Ml_sGC4       ............................................................
Oc_sGC4       ............................................................
Hc_sGC4       ............................................................
Pp_sGC2       ............................................................
Pb_sGC4       ............................................................
Hs_SGCY3      ............................................................
Hs_SGCY2      ............................................................
Ed_sGC4       ............................................................
Pb_Nitl       ............................................................
Ed_Nitl       ............................................................
Hc_Nitl       EKNEYVFIIIPTNYFPLYNWTSVLDNVYYDLSQKERDEDARKAFQSAFIVTAEVDTKLFD
Pf_Nitl2      ............................................................
Pf_Nitl       ............................................................
Ps_Nitl       ............................................................
Ca_Nitl       QSNEYGFIIIPTNYFPLYTWEGKDDDLYYNKSQKYRDEDARKAFQSAFIITADIDTSRFA
Bea_Nitl      ............................................................
Bi_sGC1       ............................................................
Bi_Nitl       ............................................................
Dg_Nitl       ............................................................
Lt_Nitl       ............................................................
Ml_NIT-like   ............................................................
Vs_Nitl       ............................................................
Oc_Nitl       ............................................................
Ocg_Nitl      ............................................................
Ocg_Nitl2     ............................................................
```

```
Hs_SGCY1     ............................................................
Ml_sGC3      ............................................................
Oc_sGC3      ............................................................
Ml_sGC1      ............................................................
Ca_sGC1      ............................................................
Pf_sGC2      ............................................................
Pb_sGC2      GHVREHRFRRTVNQRDMDQSERGERETIINVVEDIKKTVRSFECIQLTMIHFLVVDLVKL
Oc_sGC2      ............................................................
Ml_sGC2      ............................................................
Vs_sGC2      ............................................................
Hc_sGC1      ............................................................
Dg_sGC1      ............................................................
Bea_sGC1     ............................................................
Hc_sGC2      ............................................................
Oc_sGC1      ............................................................
Ml_sGC4      ............................................................
Oc_sGC4      ............................................................
Hc_sGC4      ............................................................
Pp_sGC2      ............................................................
Pb_sGC4      ............................................................
Hs_SGCY3     ............................................................
Hs_SGCY2     ............................................................
Ed_sGC4      ............................................................
Pb_Nitl      ............................................................
Ed_Nitl      ............................................................
Hc_Nitl      NFTEKVKERAKDAP.FEYEFSEGREVPLYAANYHDAVLLWSYAVEHHYNATDSTIDVPKL
Pf_Nitl2     ............................................................
Pf_Nitl      ............................................................
Ps_Nitl      ............................................................
Ca_Nitl      EFEENVKERAKQPPFDYDFDKAKRQVPLYAANYHDAVLLWAYALRNHYNSAEKSINVSAL
Bea_Nitl     ...............FNYTFPPNRKLPLYAANYHDAVLLWAYAIKHHYDPEKKGLQVEEL
Bi_sGC1      ............................................................
Bi_Nitl      ............................................................
Dg_Nitl      ............................................................
Lt_Nitl      ............................................................
Ml_NIT-like  ............................................................
Vs_Nitl      ............................................................
Oc_Nitl      ............................................................
Ocg_Nitl     ............................................................
Ocg_Nitl2    ............................................................
```

```
Hs_SGCY1     ............................................................
Ml_sGC3      ............................................................
Oc_sGC3      ............................................................
Ml_sGC1      ............................................................
Ca_sGC1      ............................................................
Pf_sGC2      ............................................................
Pb_sGC2      YKPKRKGKGAVRPLSPDGNGSVGGGSVNSWDKIQEDFNDIVDSTGKFTLLIPLPPPIPCL
Oc_sGC2      ............................................................
Ml_sGC2      ............................................................
Vs_sGC2      ............................................................
Hc_sGC1      ............................................................
Dg_sGC1      ............................................................
Bea_sGC1     ............................................................
Hc_sGC2      ............................................................
Oc_sGC1      ............................................................
Ml_sGC4      ............................................................
Oc_sGC4      ............................................................
Hc_sGC4      ............................................................
Pp_sGC2      ............................................................
Pb_sGC4      ............................................................
Hs_SGCY3     ........................................................MFCT
Hs_SGCY2     ...................MSRRKISSESFSSLGSDYLETSPEEEGECPLSRLCWNGSRS
Ed_sGC4      ............................................................
Pb_Nitl      ............................................................
Ed_Nitl      ............................................................
Hc_Nitl      YNRTYTKEDFHGIEPVTGNIKLNEFGDRVNSFVFWHLDQNSQ.WEQVGRFANGEYTVLKA
Pf_Nitl2     ............................................................
Pf_Nitl      ............................................................
Ps_Nitl      ............................................................
Ca_Nitl      YNRKFNASNFGGIEPITGDIELDEYGDRINRFKFWHLDADGQKWVEVGKYDGGEYKEIIP
Bea_Nitl     YDKTFTKEHFNGIEPITGNIKLDANGDRENSFLFWHLDHNGE.WEKVGQYVNDKYEVLVN
Bi_sGC1      ............................................................
Bi_Nitl      ............................................................
Dg_Nitl      ............................................................
Lt_Nitl      ............................................................
Ml_NIT-like  ............................................................
Vs_Nitl      ............................................................
Oc_Nitl      ............................................................
Ocg_Nitl     ............................................................
Ocg_Nitl2    ............................................................
```

```
Hs_SGCY1     ............................................................
Ml_sGC3      ............................................................
Oc_sGC3      ............................................................
Ml_sGC1      ............................................................
Ca_sGC1      ............................................................
Pf_sGC2      ............................................................
Pb_sGC2      VRSSFPDKKSLNKATFSIQSKHAFSHRMTKGMTVEGLNAEMLWTMMYIYGNLDYPVVVTL
Oc_sGC2      ............................................................
Ml_sGC2      ............................................................
Vs_sGC2      ............................................................
Hc_sGC1      ............................................................
Dg_sGC1      ............................................................
Bea_sGC1     ............................................................
Hc_sGC2      ............................................................
Oc_sGC1      ............................................................
Ml_sGC4      ............................................................
Oc_sGC4      ............................................................
Hc_sGC4      ............................................................
Pp_sGC2      ............................................................
Pb_sGC4      ............................................................
Hs_SGCY3     KLKDLKITGECPFSLLAPGQVPNESSEEAAGSSESCKATVPICQDIPEKNIQESLPQRKT
Hs_SGCY2     PPGPLEPSPAAAAAAAAPAPTPAASAAAAAATAGARRVQRRRRVNLDSLGESISRLTAPS
Ed_sGC4      ............................................................
Pb_Nitl      ............................................................
Ed_Nitl      ............................................................
Hc_Nitl      ENEIAWPNGGKSAPPYEPVCGFRNEKCPDRTSLVIGVSVGVSALLVLVAGLFVMTHMYRK
Pf_Nitl2     ............................................................
Pf_Nitl      ............................................................
Ps_Nitl      ............................................................
Ca_Nitl      TDKIAWPNGGTKAPPAEPECGFDGKGC.KSKSQTIAISVGVGAAVILVAGFIVMAYMYRK
Bea_Nitl     EADIAWPNAAKSAPQAEPECGFTGEKC.DN.TLTIAIGATTGAVAILVSGLAVMIYMYRK
Bi_sGC1      ............................................................
Bi_Nitl      ............................................................
Dg_Nitl      ............................................................
Lt_Nitl      ............................................................
Ml_NIT-like  ............................................................
Vs_Nitl      ............................................................
Oc_Nitl      ............................................................
Ocg_Nitl     ............................................................
Ocg_Nitl2    ............................................................
```

STYKc protein kinase domain

```
              1        10        20        30        40        50
Hs_SGCY1     .MYGFVNHALELLVIRNYGPEVWEDIKKEAQLDEEGQFLVRIIYDDSKTYDLVAAASKVL
Ml_sGC3      ............................................................
Oc_sGC3      ............................................................
Ml_sGC1      ............................................................
Ca_sGC1      ............................................................
Pf_sGC2      ............................................................
Pb_sGC2      FAYKSKQPNVNQESFSSYIAPFQLSISSWPRTLETNYATKTQFFRDLENLRSQIDAQQLR
Oc_sGC2      ............................................................
Ml_sGC2      ............................................................
Vs_sGC2      ............................................................
Hc_sGC1      ............................................................
Dg_sGC1      ............................................................
Bea_sGC1     ............................................................
Hc_sGC2      ............................................................
Oc_sGC1      ............................................................
Ml_sGC4      ............................................................
Oc_sGC4      ............................................................
Hc_sGC4      ............................................................
Pp_sGC2      ............................................................
Pb_sGC4      ............................................................
Hs_SGCY3     SRSRVYLHTLAESICKLIFPEFERLNVALQRTLAKHKIKESRKSLEREDFEKTIAEQAVA
Hs_SGCY2     PQTIQQTLKRTLQYYEHQVIGYRDAEKNFHNISNRCSYADHSNKEEIEDVSGILQCTANI
Ed_sGC4      ............................................................
Pb_Nitl      ............................................................
Ed_Nitl      ............................................................
Hc_Nitl      ARFEAALSAMSWKIPTSEMRDIGRDPLGSSVFGSRKSSLGTRSSLNSNPS.NLKKPTCQF
Pf_Nitl2     ............................................................
Pf_Nitl      ............................................................
Ps_Nitl      ............................................................
Ca_Nitl      ARYEAELHAMNWKIQMTEIRNINNSKLGSSIFGT.RSRLTMDSQNSSNPSAKVNGRHTQY
Bea_Nitl     VTYEAALAAMSWRIPINNVRDVARDPLGSSVFGSRRSMLATPDSSLSSQSTNLKGKTQQF
Bi_sGC1      ............................................................
Bi_Nitl      ............................................................
Dg_Nitl      ............................................................
Lt_Nitl      ............................................................
Ml_NIT-like  ............................................................
Vs_Nitl      ............................................................
Oc_Nitl      ............................................................
Ocg_Nitl     ............................................................
Ocg_Nitl2    ............................................................
```

```
              60        70        80        90        100       110
Hs_SGCY1      NLNAGEILQMFGKMFFVFCQESGYDTILRVLGSNVREFLQNLDALHDHLATIYPGMRAPS
Ml_sGC3       ............................................................
Oc_sGC3       ............................................................
Ml_sGC1       ............................................................
Ca_sGC1       ............................................................
Pf_sGC2       ............................................................
Pb_sGC2       AEEAFKAYHIMIKIIYEGVIQSLQGGSFSLVWNTLVTYETIIFSRYQLFLIESVGVMYFM
Oc_sGC2       ............................................................
Ml_sGC2       ............................................................
Vs_sGC2       ............................................................
Hc_sGC1       ............................................................
Dg_sGC1       ............................................................
Bea_sGC1      ............................................................
Hc_sGC2       ............................................................
Oc_sGC1       ............................................................
Ml_sGC4       .....................................................MTTTRFY
Oc_sGC4       ............................................................
Hc_sGC4       ............................................................
Pp_sGC2       ............................................................
Pb_sGC4       ............................................................
Hs_SGCY3      AGVPVEVIKESLGEEVFKICYEEDENILGVVGGTLKDFLNSFSTLLKQSSHCQEAGKRGR
Hs_SGCY2      LGLKFEEIQKRFGEEFFNICFHENERVLRAVGGTLQDFFNGFDALLEHIRTSFGKQATLE
Ed_sGC4       ............................................................
Pb_Nitl       ............................................................
Ed_Nitl       ............................................................
Hc_Nitl       FSKRAQYQNKVVALKTILPQYFTVNRELMIEMKTLKELRN..ENVLTVYGAIIDHDVCYL
Pf_Nitl2      ............................................................
Pf_Nitl       ............................................................
Ps_Nitl       ............................................................
Ca_Nitl       FSKRAYYQNKVVAMKVVLPQYMTINRSLMVEMKALKELRN..DNLLTVHGAISD.NLCYL
Bea_Nitl      FSRRGQYENKIVALKTVLPQYMNITRELMVQMKTLKDLRN..ENLLTVYGAIIERDISYL
Bi_sGC1       ............................................................
Bi_Nitl       ............................................................
Dg_Nitl       ............................................................
Lt_Nitl       ............................................................
Ml_NIT-like   ............................................................
Vs_Nitl       ............................................................
Oc_Nitl       ............................................................
Ocg_Nitl      ............................................................
Ocg_Nitl2     ............................................................
```

STYKc protein kinase domain

```
              120       130       140       150       160       170
Hs_SGCY1      FRCT....DAEKGKGLILHYYSEREGLQDIVIGIIKTVAQQIHGTEIDMKVIQQRNEECD
Ml_sGC3       ......................................MYGFVFDAIRLGCFKDFNKKTW
Oc_sGC3       ............................................................
Ml_sGC1       ............................................................
Ca_sGC1       ............................................................
Pf_sGC2       ............................................................
Pb_sGC2       NGTISLDIHNLFIGALKLHDYSLQFSQIYGGDTIRSKIYQSLIKYSGDLRTLLKQIRGNA
Oc_sGC2       ..MG....TSDFFDFVKLYKPNKKILGKVGKKSKGKRKGKNNGGLRPGTPESSSSDVGSV
Ml_sGC2       ..................................................MGTSYFAGSV
Vs_sGC2       ............................................................
Hc_sGC1       ............................................................
Dg_sGC1       ............................................................
Bea_sGC1      ............................................................
Hc_sGC2       ............................................................
Oc_sGC1       ............................................................
Ml_sGC4       QQTG....ILDTKLPIRLRKFLDRGSNSASLRNLCKVKAEKITWSENEVNCKMFGFMFNA
Oc_sGC4       ............................................................
Hc_sGC4       ............................................................
Pp_sGC2       ............................................................
Pb_sGC4       ............................................................
Hs_SGCY3      LEDASILCLDKEDDFLHVYYFFPKRTTSLILPGIIKAAAHVLYETEVEVSLMPPCFHNDC
Hs_SGCY2      SPSF....LCKELPEGTLMLHYFHPHHIVGFAMLGMIKAAGKKIYRLDVEVEQVANEKLC
Ed_sGC4       ............................................................
Pb_Nitl       ............................................................
Ed_Nitl       ............................................................
Hc_Nitl       VTEY....CNKGSLQDLLENDDVKLDDMLKYSLINDLAKGMRFLHSSVIESHGYLKSSNC
Pf_Nitl2      ........................................MRFLHSSVIESHGYLKSSNC
Pf_Nitl       ........................................MRFLHSSVIESHGYLKSSNC
Ps_Nitl       ............................................................
Ca_Nitl       VTEY....CNKGSLQDILENEEIKLDDTFKFSLINDLVKGMAYLHSTDIGSHGYLKSSNC
Bea_Nitl      VTEY....CSKGSLQDILENDDVKLDDMLKFSLINDLAQGLKYIHESVIDSHGYLKSSNC
Bi_sGC1       ............................................................
Bi_Nitl       ............................................................
Dg_Nitl       ............................................................
Lt_Nitl       ............................................................
Ml_NIT-like   ............................................................
Vs_Nitl       ............................................................
Oc_Nitl       ............................................................
Ocg_Nitl      ............................................................
Ocg_Nitl2     ............................................................
```

STYKc protein kinase domain

```
                180                 190       200       210       220
Hs_SGCY1     H.TQFL........IEEKESKEEDFYEDLDRFEENGTQESRISPYTFCKAFPFHIIFDRD
Ml_sGC3      Q.DISKDSGCSGEFDYSVDYDSDIFYRLADDATTKMKYTRSAVLQMFGRWYIAFIWDKWN
Oc_sGC3      ............................................................
Ml_sGC1      ............................................................
Ca_sGC1      ............................................................
Pf_sGC2      ............................................................
Pb_sGC2      HVEQSHKIALFWINIGMSYDEHIKSIQDSVAATVNEKMASMIKQAILYQYATEVVLIILC
Oc_sGC2      N.SWDR........IQEDFNDIVDSTGKDDKGGEYKRKSRNISVCIIIPILAVISYSCYH
Ml_sGC2      N.SWDR........IQEDFNDIVDSTGKDDKGSEYKRKSRNISVCIIIPILAVISYSCYH
Vs_sGC2      ............................................................
Hc_sGC1      ............................................................
Dg_sGC1      ............................................................
Bea_sGC1     ............................................................
Hc_sGC2      ............................................................
Oc_sGC1      ............................................................
Ml_sGC4      I.KLGF........YSDFEKRSWYIICTAASVPEDYDDYIDYEDQLFLELAVGLMYESYT
Oc_sGC4      ....................MELDDVLPLTSLSILSLFPFFIYFDRYNEIHSVGSTLDQI
Hc_sGC4      ............................................................
Pp_sGC2      ............................................................
Pb_sGC4      ............................................................
Hs_SGCY3     S.EFVN........QPYLLYSVHMKSTKPSLSPSKPQSSLVIPTSLFCKTFPFHFMFDKD
Hs_SGCY2     S.DVSNPGNCSCLTFLIKECENTNIMKNLPQGTSQVPADLRISINTFCRAFPFHLMFDPS
Ed_sGC4      ......................................MRERRINRVYR..PPEALADPT
Pb_Nitl      ............................................................
Ed_Nitl      ............................................................
Hc_Nitl      V.INNH........FSLKLADFSRTIFMSDMDRLSRATEEARNDRLVYR..APELLRMQM
Pf_Nitl2     V.INNH........FSLKIADFSRTIFMSDMDRLSKATEEARNERLVYR..APELLRMQC
Pf_Nitl      V.INNH........FSLKIADFSRTIFMSDMDRLSKATEEARNERLVYR..APELLRMQC
Ps_Nitl      .........................................................MQC
Ca_Nitl      I.INNL........FSLKISDFSRTIFMSDMERVSRMSAEARNERLVYR..APELLRMTT
Bea_Nitl     I.INNQ........FALKISDFSKTIFMSDMERLSRATEEAMNERLVYR..APELLRMSC
Bi_sGC1      ............................................................
Bi_Nitl      ............................................................
Dg_Nitl      ...........................MSDMERLNRANEEARNERLVYR..APELLRMQC
Lt_Nitl      ............................................................
Ml_NIT-like  ............................................................
Vs_Nitl      ............................................................
Oc_Nitl      ............................................................
Ocg_Nitl     ...........................MSDMERLNRATEAARNERLVYR..APELLRMEC
Ocg_Nitl2    ...........................MSDMERLNRATEAARNERLVYR..APELLRMEC
```

```
              230        240       250       260       270
Hs_SGCY1     LVVTQCGN.AIYRVLPQLQPGNCSLLSVFSLVRPHIDISFHGILSHI.............
Ml_sGC3      MGMIGRTPGEFFRNWSDFFEYKSQEYPLFIHDRFKIIRNLHIFYVLG.............
Oc_sGC3      ............................................................
Ml_sGC1      ..................................MLWTMMYIYGNLD.............
Ca_sGC1      ............................................................
Pf_sGC2      ............................................................
Pb_sGC2      LILSVFAVIDIRKIKQIYLYLKVSTGHLLPPCIYLVDTIPSSRDSFVWLVETGGTMKVLK
Oc_sGC2      IYQGHISHRQSRANHNLVGSSQQFSKAVEGLNAEMLWTMMYIYGNLD.............
Ml_sGC2      IYQGHISHRQSRANHNLVGSSQQFSKAVEGLNAEMLWTMMYIYGTLD.............
Vs_sGC2      ............................................................
Hc_sGC1      ............................................................
Dg_sGC1      ............................................................
Bea_sGC1     ............................................................
Hc_sGC2      ............................................................
Oc_sGC1      ............................................................
Ml_sGC4      VSKEDEQGFDL.....................AMRCWAPNMAYFGLG.............
Oc_sGC4      MPNLVRTKLNKNFFLRKPKYGTLTWNYIRSNTHMIFELCTKASEGIT.............
Hc_sGC4      ............................................................
Pp_sGC2      ............................................................
Pb_sGC4      ............................................................
Hs_SGCY3     MTILQFGNGIRRLMNRRDFQGKPNFEEYFEILTPKINQTFSGIMTML.............
Hs_SGCY2     MSVLQLGEGLRKQL.RCDTHKVLKFEDCFEIVSPKVNATFERVLLRL.............
Ed_sGC4      LIHQPST..DVYSF.AIILVEIGDRTEISPPEETIFDPLWRPELPEL.............
Pb_Nitl      ............................................................
Ed_Nitl      ............................................................
Hc_Nitl      VPCKGTKEGDMYSF.AIIVHEIIVRYGPFGLSNMDCDLTATEVINMV.............
Pf_Nitl2     IPCKGTREGDMYSF.AIIVHEIIVRYGPFGLSNMDADLTATEVINMV.............
Pf_Nitl      IPCKGTREGDMYSF.AIIVHEIIVRYGPFGLSNMDADLTATEVINMV.............
Ps_Nitl      IPCKGTREGDMYSF.AIIVHEIIVRYGPFGLSNMDADLTATEVINMV.............
Ca_Nitl      VPCQGTKEADVYSF.GIILHEVLLREGPFGLSSMECDLTSTEVIHLV.............
Bea_Nitl     VPCQGTREGDIYSF.GIVLHEILVREGPFGLSSMQSDLTATEVICMI.............
Bi_sGC1      ............................................................
Bi_Nitl      ............................................................
Dg_Nitl      IPCQGTKEGDVYSF.GIIVHEILVREGPFGLSSMECDLTAAEVMSMI.............
Lt_Nitl      ............................................................
Ml_NIT-like  ............................................................
Vs_Nitl      ............................................................
Oc_Nitl      ............................................................
Ocg_Nitl     IPCKGTKEGDMYSF.GIIVHEILVREGPFGLSNMRCDLTATEVINLV.............
Ocg_Nitl2    IPCKGTKEGDMYSF.GIIVHEILVREGPFGLSNMRCDLTATEVINLV.............
```

```
Hs_SGCY1     ............................................................
Ml_sGC3      ............................................................
Oc_sGC3      ............................................................
Ml_sGC1      ............................................................
Ca_sGC1      ............................................................
Pf_sGC2      ............................................................
Pb_sGC2      IQKDKSEALLFRMLPYSVVRELRKCRESDNLYVGSYQSATVLFADLADFQRLAARDMSPF
Oc_sGC2      ............................................................
Ml_sGC2      ............................................................
Vs_sGC2      ............................................................
Hc_sGC1      ............................................................
Dg_sGC1      ............................................................
Bea_sGC1     ............................................................
Hc_sGC2      ............................................................
Oc_sGC1      ............................................................
Ml_sGC4      ............................................................
Oc_sGC4      ............................................................
Hc_sGC4      ............................................................
Pp_sGC2      ............................................................
Pb_sGC4      ............................................................
Hs_SGCY3     ............................................................
Hs_SGCY2     ............................................................
Ed_sGC4      ............................................................
Pb_Nitl      ............................................................
Ed_Nitl      ............................................................
Hc_Nitl      ............................................................
Pf_Nitl2     ............................................................
Pf_Nitl      ............................................................
Ps_Nitl      ............................................................
Ca_Nitl      ............................................................
Bea_Nitl     ............................................................
Bi_sGC1      ............................................................
Bi_Nitl      ............................................................
Dg_Nitl      ............................................................
Lt_Nitl      ............................................................
Ml_NIT-like  ............................................................
Vs_Nitl      ............................................................
Oc_Nitl      ............................................................
Ocg_Nitl     ............................................................
Ocg_Nitl2    ............................................................
```

STYKc protein kinase domain

```
                                       280       290       300
Hs_SGCY1     ...................NTVFVL....RSKEGLLDVEKLECEDELTGTEISCLRLKGQ
Ml_sGC3      ...................MSKTSTA.....................ELEYDTPLVLQGQ
Oc_sGC3      ............................................................
Ml_sGC1      ...................YPVVVV......................SDLRIKSNESLDL
Ca_sGC1      ............................................................
Pf_sGC2      ............................................................
Pb_sGC2      AYVRFLETFYTRIAEVLESHTVNEIETAAGECLTLFCIQIKAAQREPRMFQFLHRPISAE
Oc_sGC2      ...................YPV....VVV..................SDLRIKSNESLAE
Ml_sGC2      ...................YPV....VVV..................SDLRIKSNESLAE
Vs_sGC2      ............................................................
Hc_sGC1      ............................................................
Dg_sGC1      ............................................................
Bea_sGC1     ............................................................
Hc_sGC2      ............................................................
Oc_sGC1      ............................................................
Ml_sGC4      ...................IIKFVL......................KNLFRLHEEGMSV
Oc_sGC4      ...................KLMKNE......................ETEIDLHFRLQGQ
Hc_sGC4      ............................................................
Pp_sGC2      ............................................................
Pb_sGC4      ............................................................
Hs_SGCY3     ...................NMQFVV..............RVRRWDNSVKKSSRVMDLKGQ
Hs_SGCY2     ...................STPFVI..............RTKPEASGSENKDKVMEVKGQ
Ed_sGC4      ...................N...........................EQDNDQDNKC...
Pb_Nitl      ............................................................
Ed_Nitl      ............................................................
Hc_Nitl      ...................T...........................KTDRTTPFRPEFP
Pf_Nitl2     ...................I...........................KTDRVTPFRPEFP
Pf_Nitl      ...................I...........................KTDRVTPFRPEFP
Ps_Nitl      ...................I...........................KTDRVTPFRPEFP
Ca_Nitl      ...................T...........................KTDTAQPYRPTFP
Bea_Nitl     ...................R...........................KTGTTTPYRPEFP
Bi_sGC1      ............................................................
Bi_Nitl      ............................................................
Dg_Nitl      ...................S...........................RSDLETPYRPEFP
Lt_Nitl      ............................................................
Ml_NIT-like  ............................................................
Vs_Nitl      ............................................................
Oc_Nitl      ............................................................
Ocg_Nitl     ...................T...........................KLHSQVPYRPEFP
Ocg_Nitl2    ...................T...........................KLHSQVPYRPEFP
```

STYKc protein kinase domain

HNOB

```
              310       320       330       340       350       360
Hs_SGCY1      MIYLPEADSILFLCSPSVMNLDDLTRRGLYLSDIPLHDATRDLVLLGEQFREEYKLTQEL
Ml_sGC3       MVTGMVDKSALFLCYPAVGCFLSR.RNAFYVGDLPPGPLKHTVIALICLKNSLVEHSLDL
Oc_sGC3       ............................................................
Ml_sGC1       ISTWPTTLETNYASKLEFIADLEKLRGLIDAQRLRAEEAFKAYHIMIKIIYEGVIQSLQG
Ca_sGC1       ............................................................
Pf_sGC2       ............................................................
Pb_sGC2       ISSWPRTLETNYATKTQFFRDLENLRSQIDAQQLRAEEAFKAYHIMIKIIYEGVIQSLQG
Oc_sGC2       ISSWPKTLETNYATKTQFIHDLERLRGQIDAQQLRAEEAFKAYHVMIKIIYEGVIQSLQG
Ml_sGC2       ISSWPKSLETNYATKTQFIHDLERLRGQIDAKQLRAEEAFKAYHVMIKIIYEGVIQSLQG
Vs_sGC2       ............................................................
Hc_sGC1       ............................................................
Dg_sGC1       ............................................................
Bea_sGC1      ............................................................
Hc_sGC2       ............................................................
Oc_sGC1       ............................................................
Ml_sGC4       VVEPQQVDGYIFQNNFRLQFDNTPFFRNSDEEDFSDNELDEIMPLTSLSILSLFPFFIYF
Oc_sGC4       IYVFEDTGDAIFLCSPRLESMSAC..KSTFFGDLEAHDTTKNQLVSSTLRLHLIRRLLDE
Hc_sGC4       ............................................................
Pp_sGC2       ......................MASFKSVYLSDMMAHDPIKSQVLAPSLRMHLTRRMLDE
Pb_sGC4       ........................................................MLDE
Hs_SGCY3      MIYIVESSAILFLGSPCVDRLEDFTGRGLYLSDIPIHNALRDVVLIGEQARAQDGLKKRL
Hs_SGCY2      MIHVPESNSILFLGSPCVDKLDELMGRGLHLSDIPIHDATRDVILVGEQAKAQDGLKKRM
Ed_sGC4       ....PEASKYVDLIVSCWQD.RPSARPTFEGVKKTLHIINP..YKENPVDMMMKLMEKYS
Pb_Nitl       ............................................................
Ed_Nitl       ............................................................
Hc_Nitl       FTTDVKQARVAAMCRDCWSE.DPLVRPTFPSLRSTLRSIGVE.SSGGIVGNLLSRMEKYA
Pf_Nitl2      FSTDSKQARLAAVAREAWHE.DPGMRPTFQSLRASFRSIGIE.SSGGIVGNLLSRMEKYA
Pf_Nitl       FSTDSKQARLAAVAREAWHE.DPGMRPTFQSLRASFRSIGIE.SSGGIVGNLLSRMEKYA
Ps_Nitl       FSTDSKQARLAAVAREAWHE.DPGMRPTFQSLRASFRSIGIE.SSGGIVGNLLSRMEKYA
Ca_Nitl       FTNDSKLKRAIQMANECWNE.SAHQRPTFASLRGSMKYLKPE..................
Bea_Nitl      FSTDLKIATVSALSRDCWKE.DPYQRPTIQSIRSSLRSIRTEGSSGGIVENLLSRMEKYA
Bi_sGC1       ............................................................
Bi_Nitl       ............................................................
Dg_Nitl       FSTDNKFSTISSMARSSWDE.DPLQRPTFSNIRTTLRAIRVD.TSGGIVENLLGRMEKYA
Lt_Nitl       ................................MGGQRRVREVDSSGGIVENLLGRMEKYA
Ml_NIT-like   ............................................................
Vs_Nitl       ............................................................
Oc_Nitl       ............................................................
Ocg_Nitl      FSNDPKVSRIINMAREAWSE.EAQWRPNFQSIRSTLRSIRVD.ASGGIVENLLGRMEKYA
Ocg_Nitl2     FSNDPKVSRIINMAREAWSE.EAQWRPNFQSIRSTLRSIRVD.ASGGIVENLLGRMEKYA
```

domain

```
              370
Hs_SGCY1      EILTDRLQL...................................................
Ml_sGC3       EKENMRYRE...................................................
Oc_sGC3       ............................................................
Ml_sGC1       GSFSLVWNT...................................................
Ca_sGC1       ............................................................
Pf_sGC2       ............................................................
Pb_sGC2       GSFSLVWNTLVTYETIIFSRYQLFLIESVGVMYFMNGTISLDIHNLFIGALKLHDYSLQF
Oc_sGC2       GSFSLVWNTLVTYETIIFSRYQLFLIESVGVMYFMNGTISLDIHSLFIGALKLHDYSLQF
Ml_sGC2       GSFSLVWNT...................................................
Vs_sGC2       ............................................................
Hc_sGC1       ............................................................
Dg_sGC1       ............................................................
Bea_sGC1      ............................................................
Hc_sGC2       ............................................................
Oc_sGC1       ............................................................
Ml_sGC4       DRFNEIHSV...................................................
Oc_sGC4       EREQSMLQN...................................................
Hc_sGC4       ............................................................
Pp_sGC2       ETMQGDIQA...................................................
Pb_sGC4       ETMQGDIQA...................................................
Hs_SGCY3      GKLKATLEQ...................................................
Hs_SGCY2      DKLKATLER...................................................
Ed_sGC4       KHLEMVVCE...................................................
Pb_Nitl       ............................................................
Ed_Nitl       ............................................................
Hc_Nitl       SNLEEVVEE...................................................
Pf_Nitl2      TNLEEVVEE...................................................
Pf_Nitl       TNLEEVVEE...................................................
Ps_Nitl       TNLEEVVEE...................................................
Ca_Nitl       ............................................................
Bea_Nitl      TNLEDVVEE...................................................
Bi_sGC1       ............................................................
Bi_Nitl       ............................................................
Dg_Nitl       TNLEEVVEE...................................................
Lt_Nitl       SNLEELVEE...................................................
Ml_NIT-like   ............................................................
Vs_Nitl       ............................................................
Oc_Nitl       ............................................................
Ocg_Nitl      TNLEELVEE...................................................
Ocg_Nitl2     TNLEELVEE...................................................
```

```
Hs_SGCY1      ............................................................
Ml_sGC3       ............................................................
Oc_sGC3       ............................................................
Ml_sGC1       ............................................................
Ca_sGC1       ............................................................
Pf_sGC2       ............................................................
Pb_sGC2       SQIYGGDTIRSKIYQSLIKYSGDLRTLLKQIRGNAHVEQSHKIALFWINIGMSYDEHIKS
Oc_sGC2       SQIYGGDTIRSKIYQSLIKYSGDLRTLLKQIRANAHVEQSHKIALFWINIGMSYDEHIKS
Ml_sGC2       ............................................................
Vs_sGC2       ............................................................
Hc_sGC1       ............................................................
Dg_sGC1       ............................................................
Bea_sGC1      ............................................................
Hc_sGC2       ............................................................
Oc_sGC1       ............................................................
Ml_sGC4       ............................................................
Oc_sGC4       ............................................................
Hc_sGC4       ............................................................
Pp_sGC2       ............................................................
Pb_sGC4       ............................................................
Hs_SGCY3      ............................................................
Hs_SGCY2      ............................................................
Ed_sGC4       ............................................................
Pb_Nitl       ............................................................
Ed_Nitl       ............................................................
Hc_Nitl       ............................................................
Pf_Nitl2      ............................................................
Pf_Nitl       ............................................................
Ps_Nitl       ............................................................
Ca_Nitl       ............................................................
Bea_Nitl      ............................................................
Bi_sGC1       ............................................................
Bi_Nitl       ............................................................
Dg_Nitl       ............................................................
Lt_Nitl       ............................................................
Ml_NIT-like   ............................................................
Vs_Nitl       ............................................................
Oc_Nitl       ............................................................
Ocg_Nitl      ............................................................
Ocg_Nitl2     ............................................................
```

```
Hs_SGCY1      ............................................................
Ml_sGC3       ............................................................
Oc_sGC3       ............................................................
Ml_sGC1       ..........................................................LL
Ca_sGC1       ............................................................
Pf_sGC2       ............................................................
Pb_sGC2       IQDSVAATVNEKMASMIKQAILYQYATEVVLIILCLILSVFAVIDIRKIKQIYLYLKSQS
Oc_sGC2       IQDSVAASVNEKMASMIKKAILYQYATEVVLIILCLILSVFAVIDIRKIKQIYLYLKSQS
Ml_sGC2       ........................................................LSQS
Vs_sGC2       ............................................................
Hc_sGC1       ............................................................
Dg_sGC1       ............................................................
Bea_sGC1      ............................................................
Hc_sGC2       ............................................................
Oc_sGC1       ............................................................
Ml_sGC4       ............................................................
Oc_sGC4       ............................................................
Hc_sGC4       ............................................................
Pp_sGC2       ............................................................
Pb_sGC4       ............................................................
Hs_SGCY3      ............................................................
Hs_SGCY2      ............................................................
Ed_sGC4       ............................................................
Pb_Nitl       ............................................................
Ed_Nitl       ............................................................
Hc_Nitl       ............................................................
Pf_Nitl2      ............................................................
Pf_Nitl       ............................................................
Ps_Nitl       ............................................................
Ca_Nitl       ............................................................
Bea_Nitl      ............................................................
Bi_sGC1       ............................................................
Bi_Nitl       ............................................................
Dg_Nitl       ............................................................
Lt_Nitl       ............................................................
Ml_NIT-like   ............................................................
Vs_Nitl       ............................................................
Oc_Nitl       ............................................................
Ocg_Nitl      ............................................................
Ocg_Nitl2     ............................................................
```

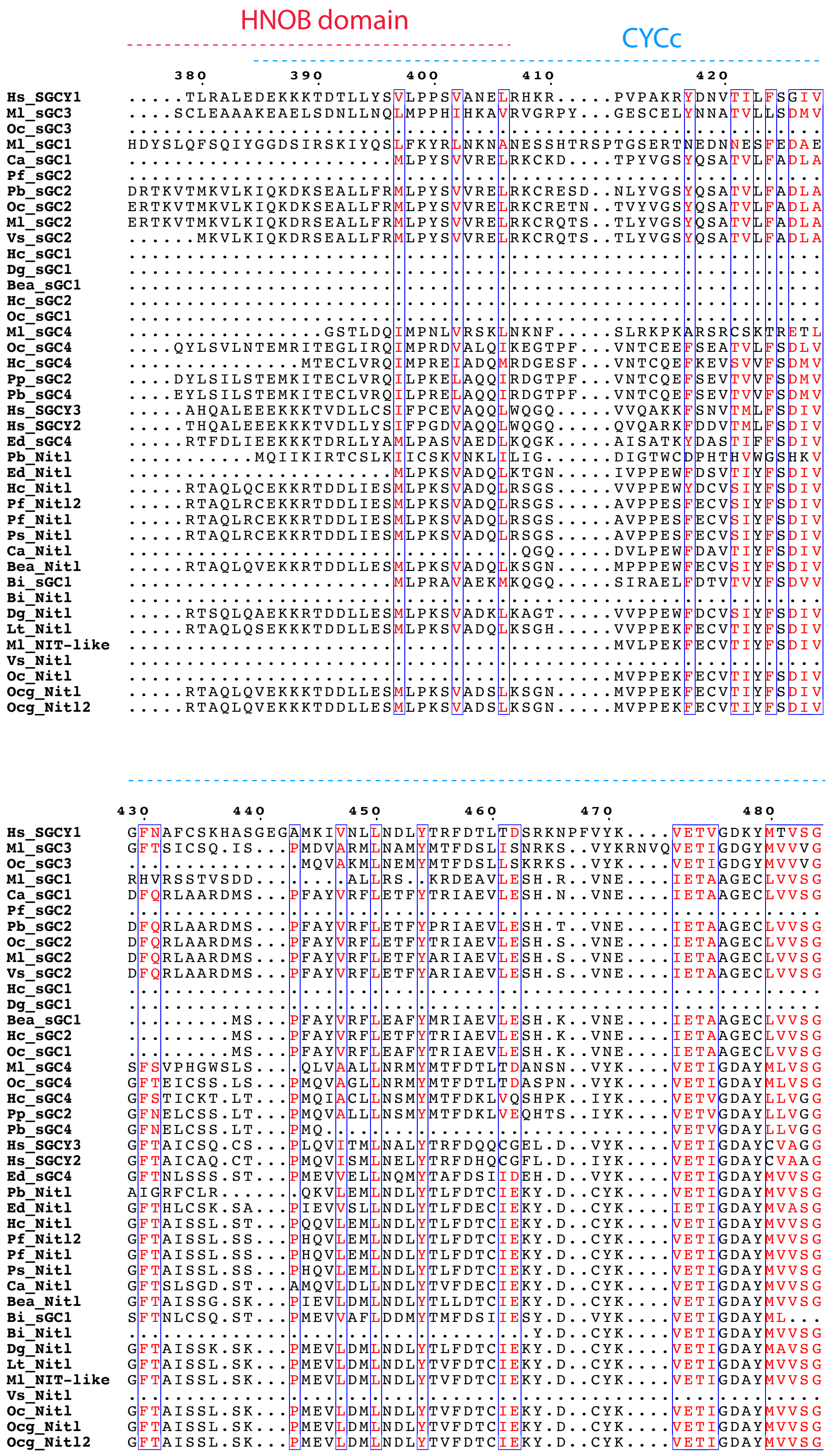
HNOB domain
CYCc
380
390
400
410
420
430
440
450
460
470
480

CYCc

Adenylyl- / guanylyl cyclase, catalytic domain

```
                   490        500       510        520        530
Hs_SGCY1     LPEPC..IHHARSICHLALDMMEIAGQVQVD...GESVQITI..GIHTGEVVTGVIGQRM
Ml_sGC3      IPNTQ..ERHAEYAADMAIDMLAGLKKVELPFLEGK.MAVKL..GINSGPIVAGVMGWKV
Oc_sGC3      IPTYQ..EKHAEYAAEMAIDMLIGLRSVELPFL.EDNMRVKI..GLNSGPVVAGVMGWKV
Ml_sGC1      LPDRSPTTKHCSEIALTALGLRSVMCRGLQLGVHGDFVCFRLIQGIASGST.........
Ca_sGC1      LPDRTT.SKHCSEIAHTALGFRSIMCRGLQLGVHGDFVCFRLIQGIATGPCAAGVIGVKL
Pf_sGC2      ............................................GIATGPCAAGIIGVKL
Pb_sGC2      LPDKT..PKHCSEIALTALGLRSVMCRGLQLGVHGDFVCFRLIQGIATGPCAAGIIGVKL
Oc_sGC2      LPDRI..PKHCSEIAMTALGLRSVMCRGLQLGVHGDFVCFRLIQGIATGPCAAGIIGVKL
Ml_sGC2      LPDRI..PKHCSEIALTALGLRSVMCRGLQLGVHGDFVCFRLIQGIATGPCAAGIIGVKL
Vs_sGC2      LPDRI..PKHCSEIALTALGLRSVMCRGLQLGVHGDFVCFRLIQGIATGPCAAGIIGVKL
Hc_sGC1      ........................MCRGLQLGVHGDFVCFRLIQGIATGPCAAGIIGVKL
Dg_sGC1      ........................MCRGLQLGVHGDFVCFRLIQGIASGPCAAGVIGVKL
Bea_sGC1     LPDRSSTSKHCSEIAHTALGLRSVMCRGLQLGVHGDFVCFRLIQGIASGPCAAGIIGVKL
Hc_sGC2      LPDRSPTSKHCAEIALTALGLRSVMCRGLQLGVHGDFVCFRLIQGIASGPCAAGIIGVKL
Oc_sGC1      LPDRSPTSKHCSEIALTALGLRSVMCRGLQLGVHGDFVCFRLIQGIASGPCAAGIIG...
Ml_sGC4      LPDRT..EHHAKHAADVAMEMVQAIQKVKINFL.KEPLSVKL..GMHSGPVVAGVVGWKV
Oc_sGC4      LPDRT..EHHAKHAADIAMEMIQAIQNVKIAFL.KEPLAVKL..GMHSGPVVAGVVGWKV
Hc_sGC4      LPIED..DNHARHAADIAIDMVQQIAEVKVDFL.TEPISIKL..GIHTGPVVA.......
Pp_sGC2      LPTKD..DNHAKYAANIAIEMVQKIQQVKVDFL.KSSISVKL..GIHTGPVVAGVVGWKV
Pb_sGC4      LPTKD..DNHAKYAANIAIEMVQKIQQVKVDFL.KSSISVKL..GIHTGPVVA...GWKV
Hs_SGCY3     LHKES..DTHAVQIALMALKMMELSDEVMSPH..GEPIKMRI..GLHSGSVFAGVVGVKM
Hs_SGCY2     LHRKS..LCHAKPIALMALKMMELSEEVLTPD..GRPIQMRI..GIHSGSVLAGVVGVRM
Ed_sGC4      VPNKNG.DKHAEEIAMMAIKIVMFCRGFRVPHRADQIVNIRA..GVHSGPVVTGVVGLKM
Pb_Nitl      LPIRNE.DRHAAQIALMSLDFLDNIRNFKIAHLPEEKLRIRI..GMHSGPVVAGIVGKKM
Ed_Nitl      LPIRNG.LRHAAQISLMSLEFLNKVESFVIRHFPEEKLKIRI..GMHSGPVCAGV.....
Hc_Nitl      LPIRNG.DRHAAQIALMSLDFLDSIRNFKIAHLPEEKLRIRI..GMHSGPVVAGIVGKKM
Pf_Nitl2     LPIQNG.DRHAAQIALMSLDFLDSISNFKIAHLPNEKLRIRI..GMHSGPVVAGIVGKKM
Pf_Nitl      LPIQNG.DRHAAQIALMSLDFLDSISNFKIAHLPNEKLRIRI..GMHSGPVVAGIVGKKM
Ps_Nitl      LPIQNG.DRHAAQIALMSLDFLDSISNFKIAHLPNEKLRIRI..GMHSGPVVAGIVGKKM
Ca_Nitl      LPIRNG.DRHAAQIALMSLDFLNNVKNFVIRHKPDAQLMIRI..GIHSGPVVAGIVGKKM
Bea_Nitl     LPIRNG.ERHAAQIGLMSLDFLDNVNNFVIGHLPNEKIKIRI..GMHSGPVVAGIVGKKM
Bi_sGC1      ............................................................
Bi_Nitl      LPVRNG.DRHAAQIALMSLNFLSCLKTFVISHLPEKKLNIRI..GMHSGPAVAGIVGKKM
Dg_Nitl      LPIRNG.DRHAAQIALMSLDFLTCIKHFVVSHLPTERLKIRI..GMHSGPVVAGIVGKKM
Lt_Nitl      LPVRNG.NRHAAQIALMSLDFLGCIKTFVISHLPEKKLMIRI..GMHSGPVVAGIVGKKM
Ml_NIT-like  LPVRNG.DRHAAQIALMSLDFLSCLNTFVISHLPDKKLNIRI..GMHSGPVVAGIVGKKM
Vs_Nitl      ...........................................................M
Oc_Nitl      LPVRNG.DRHAAQVALMSLDFLSSLKTFVISHLPDKKLNIRI..GMHSGPVVAGIVGKKM
Ocg_Nitl     LPVRNG.DRHAAQVALMSLDFLSSLNTFVISHLPDKKLNIRI..GMHSGPVVAGIVGKKM
Ocg_Nitl2    LPVRNG.DRHAAQVALMSLDFLSSLNTFVISHLPDKKLNIRI..GMHSGPVVAGIVGKKM
```

```
              540                  550      560                570      580
Hs_SGCY1     PRYCL........FGNTVNLTSRTETTGEKGKINV.......SEYTYRCLMSPENSDPQF
Ml_sGC3      PRYGV........FGDTVNVVNVLEST.............................SKNF
Oc_sGC3      PRYGV........FGDTVNVVNVLESTSKPNRIHI.......SES...............
Ml_sGC1      ............................................................
Ca_sGC1      PRFCV........FGNTVNTAARMKSTCLGMRIQI.......TEACKYVL......EEEF
Pf_sGC2      PRFCV........FGDTINTAARMKSTAIGMRIQI.......TDVCCDIL......EEEF
Pb_sGC2      PRFCV........FGDTINTAARMKSTAIGMRIQI.......TDVCCDIL......EEEF
Oc_sGC2      PRFCV........FGDTVNTAARMKSTAIGMRIQI.......TDVCSEVL......EEEF
Ml_sGC2      PRFCV........FGDTVNTAARMKSTAIGMRIQI.......TDVCSEVL......EDEF
Vs_sGC2      PRFCV........FGDTVNTAARMKSTAIGMRIQI.......TDVCSEVL......EDEF
Hc_sGC1      PRFCV........FGDTINTAARMKSTAIGMRIQI.......TDVCCDIL......EDDF
Dg_sGC1      PRFCV........FGDTVNTAARMKSSCLGMRIQI.......TDICSVIL......EEGF
Bea_sGC1     PRFCV........FGDTVNTAARMKSTCLGMRIQI.......TDICSIIL......EEGF
Hc_sGC2      PRFCV........FGDTVNTAARMKSTCLGMRIQI.......TDICSFIL......EEGF
Oc_sGC1      ............................................................
Ml_sGC4      PRYCL........FGDTVNTASRMESASEALKIHI.......SDSTNEHL....KRYGNY
Oc_sGC4      PRYCL........FGDTVNTASRMETASEALKIHI.......SDSTNEHL....KRYGNY
Hc_sGC4      ............................................................
Pp_sGC2      PRYCL........FGDTVNTASRMETSSEALKIHI.......SDATNECL....KRYGNY
Pb_sGC4      PRFCLSLYLCLSVFGDINTWCDPRQVSMSPMSVCLFVCLSLSLSTPFLQVWWDGRCRGNY
Hs_SGCY3     PRYCL........FGNNVTLANKFESCSVPRKINV.......SPTTYRLL....KDCPGF
Hs_SGCY2     PRYCL........FGNNVTLASKFESGSHPRRINV.......SPTTYQLL....KREESF
Ed_sGC4      PRFCL........FGDTVNMASRMESTGEALKIQV.......SPTTHRLL....KDREDF
Pb_Nitl      PRYCL........FGDTVNTASRMESNGEAEKIQM.......SSQSHDIL....KINGGF
Ed_Nitl      ............................................................
Hc_Nitl      PRYCL........FGDTVNTASRMESNGEALKIQM.......SLQSHDIL....NIHGGF
Pf_Nitl2     PRYCL........FGDTVNTASRMESNGEALKIQM.......SSQSHDIL....KIHGGF
Pf_Nitl      PRYCL........FGDTVNTASRMESNGEALKIQM.......SSQSHDIL....KIHGGF
Ps_Nitl      PRYCL........FGDTVNTASRMESNGEALKIQM.......SSQSHDIL....KIHGGF
Ca_Nitl      PRYCL........FGDTVNTASRMESNGQALKIHM.......SEQTYNIL....IKHGGF
Bea_Nitl     PRYCL........FGDTVNTASRMESNGEALKIQM.......SEQSYKIL....DNHGGF
Bi_sGC1      ............................................................
Bi_Nitl      PRYCL........FGDTVNTASRMESNGEPEKIQM.......SSQSYSIL....SSHGGF
Dg_Nitl      PRYCL........FGDTVNTASRMESNGEPERIQM.......SEQSYNIL....ASHGGF
Lt_Nitl      PRYCL........FGDTVNTASRMESNGEALKIQM.......SSQSYGIL....SEHGGF
Ml_NIT-like  PRYCL........FGDTVNTASRMESNGEAKKIQM.......STQSYGIL....SNHGGF
Vs_Nitl      PRYCL........FGDTVNTASRMESNGEAKKIQM.......STQSYGIL....SNHGGF
Oc_Nitl      PRYCL........FGDTVNTASRMESNGMAEKIQM.......SSQSYNIL....ANHGGF
Ocg_Nitl     PRYCL........FGDTVNTSSRMESNGIAGKIQM.......SSQSYSIL....SNHGGF
Ocg_Nitl2    PRYCL........FGDTVNTSSRMESNGIAGKIQM.......SSQSYSIL....SNHGGF
```

```
                     590       600
Hs_SGCY1       HLEHR.GPVSMKGKKEPMQVWFL...SRKNT.............................
Ml_sGC3        ISR...............TYWLC...GRRTSDTNPFENEEAEDEALP.............
Oc_sGC3        ............................................................
Ml_sGC1        ............................................................
Ca_sGC1        LISKR.GVIYVKGKGEMETHWLI...GVRNN.............................
Pf_sGC2        LISKR.GTIFVKGKGEMETHWLI...GNRNS..........NEDINN.............
Pb_sGC2        LISKR.GTIFVKGKGEMETHWLI...GSRNSNEDMNSYSNNNNNNNNNGGGNKSGSGGGR
Oc_sGC2        IVSKR.GIIFVKGKGEMETHWLI...GVRNS...........NEDLS.............
Ml_sGC2        IVSKR.GVIFVKGKGEMETYWLI...GVRNSTEDLTSYNNNSNNNNN.............
Vs_sGC2        IVSKR.GVIFVK................................................
Hc_sGC1        LIAKR.GTIFVKGKGEMETHWLI...GNRTS.............................
Dg_sGC1        LVTKR.GTIFVKGKGEMETHWLI...GERN..............................
Bea_sGC1       VVTKR.GTIFVKGKGEMETHWLI...SERNS.............................
Hc_sGC2        VISRR.GIIFVKGKGELETHWLI...GERNS.............NDD.............
Oc_sGC1        ............................................................
Ml_sGC4        ITRQR.GWHSHKGKGQLPTYWLC...GQGDNILPSYTQTELMEEEAK.............
Oc_sGC4        ITRQR.GWHAHKGKGQLPTYWLC...GQGDTILASYTKNELMEEEAK.............
Hc_sGC4        ............................................................
Pp_sGC2        ITRQR.GWHDHKGKGQLPTYWLC...GQGDTLLTSYTKEELTEEEAK.............
Pb_sGC4        ITRQR.GWHDHKGKGQLPTYWLC...GQGDT.............................
Hs_SGCY3       VFTPR.......SREELPPNFPS.....................................
Hs_SGCY2       TFIPR.......SREELPDNFPK.....................................
Ed_sGC4        RFEYR.GKVEVKGKGTQETHWLL...GRDN..............................
Pb_Nitl        VTESR.GLVEMKVLEMLNDLYTLFDTCIEKYDCYKVETIGDAYMVVS.............
Ed_Nitl        ............................................................
Hc_Nitl        VTESR.GLVEMKGKGKVPTFWLK...GVEND.............................
Pf_Nitl2       VTECR.GLVEMKGKGKVETFWLK...GVENN.............................
Pf_Nitl        VTECR.GLVEMKGKGKVETFWLK...GVENN.............................
Ps_Nitl        VTECR.GLVEMKGKGKVETFWLK...GVENN.............................
Ca_Nitl        VIEYR.GLVEMKGKGKLPTYWLQ...GLDE..............................
Bea_Nitl       IIEAR.GLIEMKGKGLVQTYWLQ...GVDKA.............................
Bi_sGC1        ............................................................
Bi_Nitl        IIDPREGLVDMKGKGKQQTYWLQ...GVEKD.............................
Dg_Nitl        VIEPR.GLVEMKGKGKIPTYWLQ...SIEKD.............................
Lt_Nitl        EIDPREGLIEMKGKGLQQTYWLQ...GVEKD.............................
Ml_NIT-like    VIQPRDGLVDMKGKGKQQTYWLQ...GVEKD.............................
Vs_Nitl        VIQPRDGLVDMKGKGKQQTYWLQ...GVEKD.............................
Oc_Nitl        VIEPRDGLVDMKGKGLQQTYWLH...GVEKY.............................
Ocg_Nitl       IIEPRDGLVDMKGKGLQQTYWLQ...GVEKY.............................
Ocg_Nitl2      IIEPRDGLVDMKGKGLQQTYWLQ...GVEKY.............................
```

```
Hs_SGCY1       ............................................................
Ml_sGC3        ............................................................
Oc_sGC3        ............................................................
Ml_sGC1        ............................................................
Ca_sGC1        ............................................................
Pf_sGC2        ............................................................
Pb_sGC2        NKKKQSNWSMESAARGVVRQIALTNPILVITHNLCSNPPFTFVKEAGVTEGLGGASADDG
Oc_sGC2        ............................................................
Ml_sGC2        ............................................................
Vs_sGC2        ............................................................
Hc_sGC1        ............................................................
Dg_sGC1        ............................................................
Bea_sGC1       ............................................................
Hc_sGC2        ............................................................
Oc_sGC1        ............................................................
Ml_sGC4        ............................................................
Oc_sGC4        ............................................................
Hc_sGC4        ............................................................
Pp_sGC2        ............................................................
Pb_sGC4        ............................................................
Hs_SGCY3       ............................................................
Hs_SGCY2       ............................................................
Ed_sGC4        ............................................................
Pb_Nitl        ............................................................
Ed_Nitl        ............................................................
Hc_Nitl        ............................................................
Pf_Nitl2       ............................................................
Pf_Nitl        ............................................................
Ps_Nitl        ............................................................
Ca_Nitl        ............................................................
Bea_Nitl       ............................................................
Bi_sGC1        ............................................................
Bi_Nitl        ............................................................
Dg_Nitl        ............................................................
Lt_Nitl        ............................................................
Ml_NIT-like    ............................................................
Vs_Nitl        ............................................................
Oc_Nitl        ............................................................
Ocg_Nitl       ............................................................
Ocg_Nitl2      ............................................................
```

```
Hs_SGCY1      ............................................................
Ml_sGC3       ........VAVAKQPVTKTKKTTVP...................................
Oc_sGC3       ............................................................
Ml_sGC1       ............................................................
Ca_sGC1       .........MTEEEMSVYAGNKMSST..................................
Pf_sGC2       ........YSNNNNSGGNTGHKQSGSR.................................
Pb_sGC2       KEGHKSKSKSSKSNAFQFLTTSVNIIFSLHHLSLAPQPISLYITQVSGSCEWTRLRSRSS
Oc_sGC2       ........NYNSGNNNSASKNTS.....................................
Ml_sGC2       ........NNNNNNNNNNNNNSGNKTSNR...............................
Vs_sGC2       ............................................................
Hc_sGC1       ...........NDEMNNYNNGSSGDSRQGATDPGREPVR.....................
Dg_sGC1       ............................................................
Bea_sGC1      ............................................................
Hc_sGC2       ........IQRLTNKSTITNHASAPAAA................................
Oc_sGC1       ............................................................
Ml_sGC4       ........KQVNQVLEQVNEGAQLSADVLNQLSNNP........................
Oc_sGC4       ........KQVNQVLEQVNEGALSVDVLQQLSNNPALQRQIN..................
Hc_sGC4       ............................................................
Pp_sGC2       ........KQVNDVLEQLPDGSTAAAEMIQQMGGFNPALQR...................
Pb_sGC4       ............................................................
Hs_SGCY3      ..........EIPGICHFLDAYQQG...................................
Hs_SGCY2      ..........EIPGICYFLEVRTGPKP.................................
Ed_sGC4       .........LSISNCLSPASHKRIFKGALP..............................
Pb_Nitl       ........GLPIRNEDRHAAQIALMSLDFLDNIRNFKIAHLPEEKLRIRIGMHSDNLTSR
Ed_Nitl       ............................................................
Hc_Nitl       .........INLNNPRHFTDQAALL...................................
Pf_Nitl2      .........INLTNPRTFTDQAVLA...................................
Pf_Nitl       .........INLTNPRTFTDQAVLA...................................
Ps_Nitl       .........INLTNPRTFTDQAVLA...................................
Ca_Nitl       ............................................................
Bea_Nitl      .........LNLTNPRQFTDQTTQK...................................
Bi_sGC1       ............................................................
Bi_Nitl       .........LNIDNPRHFTDRTALQ...................................
Dg_Nitl       .........LHVLNPRQFTDQATAL...................................
Lt_Nitl       .........LNVDNPRHFTDHTALL...................................
Ml_NIT-like   .........LNIDNPRHFTDKTALL...................................
Vs_Nitl       .........LNIDNPRHFTDKTALL...................................
Oc_Nitl       .........LNVDNPRQFTDKATLM...................................
Ocg_Nitl      .........LNIGNPRHFTDKTALL...................................
Ocg_Nitl2     .........LNIGNPRHFTDKTALL...................................
```

```
                                                  610
                                                    .
Hs_SGCY1      ...................................GTEETKQDDD...............
Ml_sGC3       ..................TDMGLDVMVDPESSSVISAEGVRSVEERWKIVVEKIRKFEKE
Oc_sGC3       ............................................................
Ml_sGC1       ............................................................
Ca_sGC1       ..................QKVQDIPNEDQVTSSKSSNKKQKDDKSKFSMNSKSLGPFS..
Pf_sGC2       ..................SQKKQSNWSMEEGLGGAGDEGKGGHKSKAKSSRSNG......
Pb_sGC2       IDQSEDCVTIPSGRPTTAPDTSQVSFRTSRQNSAAAGSRDFINLQEQSTKNVSMVNRSTH
Oc_sGC2       ..................NRKKASNWSGTDQTNGVGAEDSKSKSKTKPGRSNGGVNGR..
Ml_sGC2       ..................KQPVRTTWPGQPTSSTHTNEEAKSKNKPKAGHSNGGLNSR..
Vs_sGC2       ............................................................
Hc_sGC1       ..................KAKKQSSSWSIEEGLRGGATDDLKDGHKSKPKSDRSPKYG..
Dg_sGC1       ............................................................
Bea_sGC1      .......................................NHDYDDIPRHAGNSNQNKS..
Hc_sGC2       ..................RQKGTTNNKQEVPLTPMQGAGSGAVNSPAATAAVSGPNVSTS
Oc_sGC1       ............................................................
Ml_sGC4       ..................ALQRQINPLQKVAELSAESRELNNDDEREHKNRKQTFNVRNV
Oc_sGC4       ..................PLQKVAELGKEEQGVEEGARKIKEEEENGRKKKKGAIKIENV
Hc_sGC4       ............................................................
Pp_sGC2       ..................QLQPGGSTSSKPNVKTPGGGGPLNKIHPKISPSEVGSVLAAP
Pb_sGC4       ............................LLSSYTKEELTEEEAKKQVNDVLEQVGH....
Hs_SGCY3      ..................TNSKPCFQKKDVEDGNANFLGKASGID...............
Hs_SGCY2      ..................PKPSLSSSRIKKVSYNIGTMFLRETSL...............
Ed_sGC4       ..................KPKPLTPTPAMLALETPDTSEPAANSTPTPPAEPTVPILPPP
Pb_Nitl       ILAHRYPSGPVVAGIVGKKMPRYCLFGDTVNTASRMESNGEVNNRIFKKLKNQKQIISCF
Ed_Nitl       ............................................................
Hc_Nitl       ..................KK.G..........SYGDQPG.AGFMNIVNIYNPSEEKTH.D
Pf_Nitl2      ..................KRSG..........VYSDGGAPPNYMNIVNIYNPDYDKTHND
Pf_Nitl       ..................KRSG..........VYSDGGAPPNYMNIVNIYNPDYDKTHND
Ps_Nitl       ..................KRSG..........VYSDGGAPPNYMNIVNIYNPDYDKTHND
Ca_Nitl       ............................................................
Bea_Nitl      ..................RKK.......................................
Bi_sGC1       ............................................................
Bi_Nitl       ..................KRESNAVTTHATNLVYTVDSNSKKNTVKSSSSPNDMVNYNNL
Dg_Nitl       ..................KKNSFVEDRKSNNKHLYNNLNHENNKHHKDTHYKEFP.....
Lt_Nitl       ..................KRDTSSSQ......ILIDSSKEKKQSIKSKSVVPDIINFNNL
Ml_NIT-like   ..................KRESNSQF......YTDTDTTKKQTVLPKTPQSKDMVKLQQS
Vs_Nitl       ..................KRESNSQF......YTDTDTTKKQTVLPKTPQSKDMVNFNNL
Oc_Nitl       ..................KKESNNQFYL................................
Ocg_Nitl      ..................KKEASNQI......FLVGDSSKEGGKSPQ..PSPEMVNYNNL
Ocg_Nitl2     ..................KKEASNQI......FLVGDSSKEGGKSPQ..PSPEMVNYNNL
```

```
Hs_SGCY1      ............................................................
Ml_sGC3       SKICSIC.....................................................
Oc_sGC3       ............................................................
Ml_sGC1       ............................................................
Ca_sGC1       ............................................................
Pf_sGC2       ............................................................
Pb_sGC2       WKIASAGGRPEKSCTDSKLDISKILAQVELKNKMRRDEAFRPEYSLMELERIKAMNMWLS
Oc_sGC2       ............................................................
Ml_sGC2       ............................................................
Vs_sGC2       ............................................................
Hc_sGC1       ............................................................
Dg_sGC1       ............................................................
Bea_sGC1      ............................................................
Hc_sGC2       ASATTAAVQHSPV...............................................
Oc_sGC1       ............................................................
Ml_sGC4       STICTIL.....................................................
Oc_sGC4       SAICNIL.....................................................
Hc_sGC4       ............................................................
Pp_sGC2       PTPIRASRQDELSCSRLCSIM.......................................
Pb_sGC4       ............................................................
Hs_SGCY3      ............................................................
Hs_SGCY2      ............................................................
Ed_sGC4       PYSTSPSRERSSGSTSSRDKLLV.....................................
Pb_Nitl       KLCELVNIEKTLSXLQYKN.........................................
Ed_Nitl       ............................................................
Hc_Nitl       KHLQ........................................................
Pf_Nitl2      KHLQ........................................................
Pf_Nitl       KHLQ........................................................
Ps_Nitl       KHLQ........................................................
Ca_Nitl       ............................................................
Bea_Nitl      ............................................................
Bi_sGC1       ............................................................
Bi_Nitl       NHVDSKNHKVHLNNKEFQ..........................................
Dg_Nitl       ............................................................
Lt_Nitl       NHSDKKSHKVHLNNKEFRS.........................................
Ml_NIT-like   ............................................................
Vs_Nitl       NHVDSKNHKVHLNNKEFQ..........................................
Oc_Nitl       ............................................................
Ocg_Nitl      NHVDSKDHKVHLNNCKEFP.........................................
Ocg_Nitl2     NHVDSKDHKVHLNNCKEFP.........................................
```

```
Hs_SGCY1      ..............................
Ml_sGC3       ..............................
Oc_sGC3       ..............................
Ml_sGC1       ..............................
Ca_sGC1       ..............................
Pf_sGC2       ..............................
Pb_sGC2       LHLVAMQFLNMYHLLHAIYNVVSFSSNLLI
Oc_sGC2       ..............................
Ml_sGC2       ..............................
Vs_sGC2       ..............................
Hc_sGC1       ..............................
Dg_sGC1       ..............................
Bea_sGC1      ..............................
Hc_sGC2       ..............................
Oc_sGC1       ..............................
Ml_sGC4       ..............................
Oc_sGC4       ..............................
Hc_sGC4       ..............................
Pp_sGC2       ..............................
Pb_sGC4       ..............................
Hs_SGCY3      ..............................
Hs_SGCY2      ..............................
Ed_sGC4       ..............................
Pb_Nitl       ..............................
Ed_Nitl       ..............................
Hc_Nitl       ..............................
Pf_Nitl2      ..............................
Pf_Nitl       ..............................
Ps_Nitl       ..............................
Ca_Nitl       ..............................
Bea_Nitl      ..............................
Bi_sGC1       ..............................
Bi_Nitl       ..............................
Dg_Nitl       ..............................
Lt_Nitl       ..............................
Ml_NIT-like   ..............................
Vs_Nitl       ..............................
Oc_Nitl       ..............................
Ocg_Nitl      ..............................
Ocg_Nitl2     ..............................
```
